# Absence of the primary cilia formation gene Talpid3 impairs muscle stem cell function

**DOI:** 10.1038/s42003-023-05503-9

**Published:** 2023-11-04

**Authors:** Victor Martinez-Heredia, Danielle Blackwell, Sujith Sebastian, Timothy Pearson, Gi Fay Mok, Laura Mincarelli, Charlotte Utting, Leighton Folkes, Ernst Poeschl, Iain Macaulay, Ulrike Mayer, Andrea Münsterberg

**Affiliations:** 1 School of Biological Sciences, University of East Anglia, Norwich Research Park, Norwich, UK; 2 The Earlham Institute, Norwich Research Park, Norwich, UK; 3 Present Address: Barcelona Institute for Science & Technology, Center for Genome Regulation CRG, Dr Aiguader 88, 08003 Barcelona, Spain; 4 Present Address: Alberta Children’s Hospital Research Institute (ACHRI), University of Calgary, Calgary, AB Canada; 5 Present Address: Clinical Biotechnology Center, NHSBS, Bath, UK; 6 Present Address: Wellcome Sanger Institute, Wellcome Trust Genome Campus, Hinxton, Saffron Walden, CB10 1RQ UK

**Keywords:** Genetics, Stem cells, Muscle stem cells

## Abstract

Skeletal muscle stem cells (MuSC) are crucial for tissue homoeostasis and repair after injury. Following activation, they proliferate to generate differentiating myoblasts. A proportion of cells self-renew, re-enter the MuSC niche under the basal lamina outside the myofiber and become quiescent. Quiescent MuSC have a primary cilium, which is disassembled upon cell cycle entry. Ex vivo experiments suggest cilia are important for MuSC self-renewal, however, their requirement for muscle regeneration in vivo remains poorly understood. Talpid3 (TA^3^) is essential for primary cilia formation and Hedgehog (Hh) signalling. Here we use tamoxifen-inducible conditional deletion of TA^3^ in MuSC (iSC-KO) and show that regeneration is impaired in response to cytotoxic injury. Depletion of MuSC after regeneration suggests impaired self-renewal, also consistent with an exacerbated phenotype in TA^3iSC-KO^ mice after repeat injury. Single cell transcriptomics of MuSC progeny isolated from myofibers identifies components of several signalling pathways, which are deregulated in absence of TA^3^, including Hh and Wnt. Pharmacological activation of Wnt restores muscle regeneration, while purmorphamine, an activator of the Smoothened (Smo) co-receptor in the Hh pathway, has no effect. Together, our data show that TA^3^ and primary cilia are important for MuSC self-renewal and pharmacological treatment can efficiently restore muscle regeneration.

## Abstract (web summary)

The Talpid3 gene, essential for primary cilia formation, is required for skeletal muscle stem cell (MuSC) self-renewal and loss of Talpid3 in MuSCs deregulates hedgehog and Wnt signalling pathways.

## Introduction

Primary cilia are non-motile microtubule-based structures found on many cells. They serve as a cellular antenna for multiple signalling pathways and are implicated in numerous developmental disorders. The importance of primary cilia for Sonic hedgehog (Hh) signalling in vertebrates was uncovered in a mouse mutagenesis screen, which identified genes encoding intraflagellar transport (IFT) proteins^1^ required to build and maintain primary cilia. The sequestration of Hh pathway components into the cilium regulates the down-stream processing of GLI effector proteins, with GLI1/2 acting as transcriptional activators and GLI3 acting predominantly as a transcriptional repressor^2–5^. Smoothened (Smo), a seven-pass transmembrane protein and Hh co-receptor, moves into the ciliary membrane depending on pathway activity. A conserved ciliary localisation motif has been identified^6,7^.

Many ciliary proteins have now been characterised and mutations in some of these are associated with diseases in human, collectively termed ciliopathies^8^. Thus, cilia are important for human health, however, their role in skeletal muscle remains enigmatic^9,10^. Talpid3 (TA^3^) is a critical component of primary cilia. The protein is located to the distal end of centrioles and necessary for docking of the basal body with the apical cell membrane and thus, for primary cilia formation^11,12^. The TA^3^ gene was first identified in a spontaneous chicken mutant and shown to be essential for embryonic patterning and myogenesis^13^. The defects seen in the original TA^3^ mutant include features that can be attributed to abnormal Hh signalling, such as limb and neural tube defects^14^. These were phenocopied in a mouse knock-out where exons essential for TA^3^ protein function were deleted^11,15^.

The Hh pathway is the most investigated cilia-related signalling pathway, however, there is extensive crosstalk between Wnt and Hh signalling pathways, which share common regulators—including GSK3β, CK1α and Smoothened (Smo). Both pathways are important in embryonic myogenesis^16–18^. GLI proteins have positive and negative regulatory functions controlling expression of the myogenic regulatory factors (MRF), Myf5 and MyoD during development^19–23^. Shh also promotes the proliferation and differentiation of cultured myogenic cells^24,25^. Wnt signalling is active during embryonic myogenesis and it has multiple functions during the muscle regenerative cycle (reviewed in ref. ^26^). Its timely activation is important to control muscle progenitor cell proliferation and differentiation^27–30^. A transient activation of Wnt/β-catenin (canonical) signalling in myoblasts is critical, while constitutive β-catenin activation leads to a prolonged regenerative response^28^. Interestingly, both MuSC-specific β-catenin gain- or loss-of-function mutations perturb muscle regeneration, suggesting appropriate levels of signalling are essential for MuSC function^30^.

In addition, Wnt and Shh signalling pathways are linked to primary ciliogenesis and depending on the context, they act cooperatively or antagonistically^31–34^. Quiescent muscle stem cells (MuSC) have a primary cilium, which is disassembled upon activation and cell cycle entry and reassembled in self-renewing MuSC^35^. Knock-down of IFT88 in C2C12 myoblasts leads to ablation of ciliogenesis and reduced expression of p27, a marker for quiescence^36^. In mice, conditional deletion of IFT88 in MuSC, impairs recovery of overall muscle strength and alters the expression of cell-cycle-related genes. This group also found that primary cilia are lost during aging, suggesting a possible role of cilia in age-related muscle loss^37^. Repression of Hh signalling seems to be important in maintaining MuSC quiescence in G_0_^37,38^. This requires the repressor form of GLI3, since conditional deletion of GLI3, or inhibition of its processing into GLI3R, leads to cell cycle entry^39^. Together this suggests that Hh signalling regulates MuSC activation and proliferation. However, the role of primary cilia as a nexus for signalling pathways to facilitate MuSC self-renewal after skeletal muscle regeneration in response to injury has not yet been investigated in vivo.

Here we examine the role of primary cilia as a nexus for signalling pathways to regulate MuSC behaviour during regeneration in response to injury. We use the floxed allele of TA^3,^^11,15^, to generate an inducible conditional knock-out in MuSC, termed TA^3iSC-KO^, using PAX7-Cre^ERT2^. In adult mice Pax7, and thus Cre^ERT2^, is specifically expressed in MuSC. Recombination of the floxed TA^3^ locus is triggered in these cells after tamoxifen administration^40,41^. Using cardiotoxin (ctx)-induced injury, we show that TA^3^ function is required for efficient muscle repair. In TA^3iSC-KO^ mutants, fibre diameters were smaller and the number of Pax7-positive MuSC was reduced after injury-induced regeneration. Repeat injury enhanced the phenotype, together suggesting that MuSC self-renewal is affected.

We used two reporter alleles, Pax7-ZsGreen^42^ or Rosa-fl-stop-fl-Td-Tomato to FACS isolate MuSC for molecular analysis. Pax7-ZsGreen-positive quiescent MuSC were isolated at day 0. In addition, Td-Tomato-positive MuSC and their progeny were isolated from cultured myofibers at 72 h. Transcriptomics revealed effects on ECM-related genes and de-regulated signalling pathways in absence of TA^3^; in particular, many Wnt ligands were downregulated. Consistent with the latter, we show that muscle regeneration was rescued by co-injection of 6-bromoindirubin-3′-oxime (BIO), an activator of β-catenin-dependent Wnt signalling^43^, but not by purmorphamine (Pur), an activator of the Hh co-receptor, Smo^44^. Myofiber diameter and the number of Pax7-positive (Pax7^+ve^) MuSC were restored by BIO co-injection. Therefore, we propose that during the regenerative cycle modulation of signalling via the primary cilium promotes MuSC efficient self-renewal. This might be important in muscle disease or in ageing when MuSC function declines.

## Results

### TA^3^ is necessary for primary cilia formation in MuSCs

To examine the requirement of Talpid3 (TA^3^) for primary cilia formation in MuSCs, we used a conditional allele of the TA^3^ gene^15^ with the Pax7^CreERT2^ driver^40,41,45^. After only three consecutive tamoxifen injections, we observed a recombination efficiency of 92-94% in myofibers isolated from TA^3iSC-KO^ mutants or controls. MuSC were identified using Pax7-ZsGreen fluorescence and recombination was confirmed using the Td-Tomato reporter (Supplementary Fig. 1). In all experiments we used a well-established regime of tamoxifen injections^40,45^ prior to myofiber isolation. Thus, homozygous TA^3flox/flox^/Pax7^CreERT2/+^ animals (TA^3iSC-KO^) lack expression of the gene in >92% of MuSC. Heterozygous TA^3flox/+^ litter mates or animals lacking Pax7^CreERT2^ were used as controls.

**Fig. 1:**
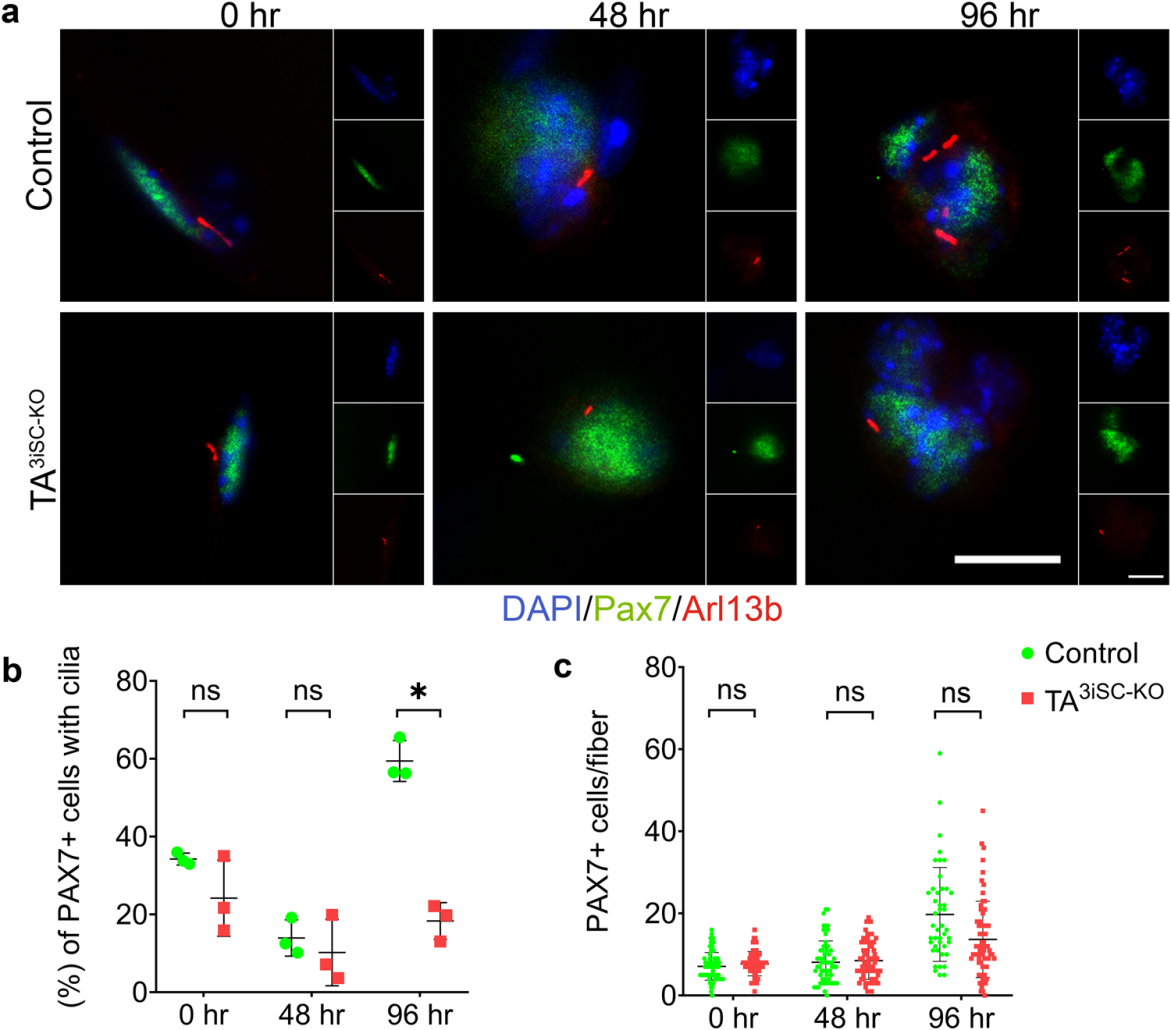
Cilia assembly in MuSCs requires TA^3^. **a** Representative images of EDL muscle fibres from TA^3iSC-KO^ and control mice at 0, 48 and 96 h. Immunofluorescence detects Pax7 expressing MuSCs (green) and primary cilia using Arl13b (red), DAPI (blue) stains nuclei (scale bars = 10 µm). **b** The percentage of Pax7^+ve^ cells positive for Arl13b in fibres from TA^3iSC-KO^ and control mice, *n* = 3 mice, >10 fibres per mouse, *p < 0.05 two-tail *t* test, mean and SD are shown. **c** The average number of Pax7^+ve^ cells per fibre after 0, 48 and 96 h in culture, control (green) and TA^3iSC-KO^ (red), *n* = 3 mice, >10 fibres per mouse, *p < 0.05 unpaired, two-tailed *t* test, mean and SD are shown. After 96 h, Pax7^+ve^ cells are reduced on mutant fibres.

To detect primary cilia in MuSC, myofibers were isolated, cultured for 0, 48 and 96 h and immunostained using Arl13b and Pax7 antibodies (Fig. 1a). At 0 and 48 h, the percentage of Pax7^+ve^ cells with cilia was similar in TA^3iSC-KO^ and control fibres. This was expected, as primary cilia are disassembled only upon entry into the cell cycle^35^, which occurs >40 h after activation. After 96 h in culture, MuSC have undergone multiple rounds of cell division, a proportion of cells re-express Pax7, re-enter the stem cell niche and re-assemble a cilium. At this point 60% of Pax7 expressing cells had a primary cilium in control myofibers, similar to previous reports^35,39^. In TA^3iSC-KO^ mutants, there were fewer Pax7^+ve^ cells with a primary cilium, and the number of Pax7^+ve^ cells was reduced in TA^3iSC-KO^ mutant fibres (Fig. 1b, c and Supplementary Data 1). Remaining Pax7^+ve^ cells with a primary cilium are most likely the progeny of MuSC that have escaped recombination.

### MuSCs lacking TA^3^ have a proliferation defect

**Fig. 2:**
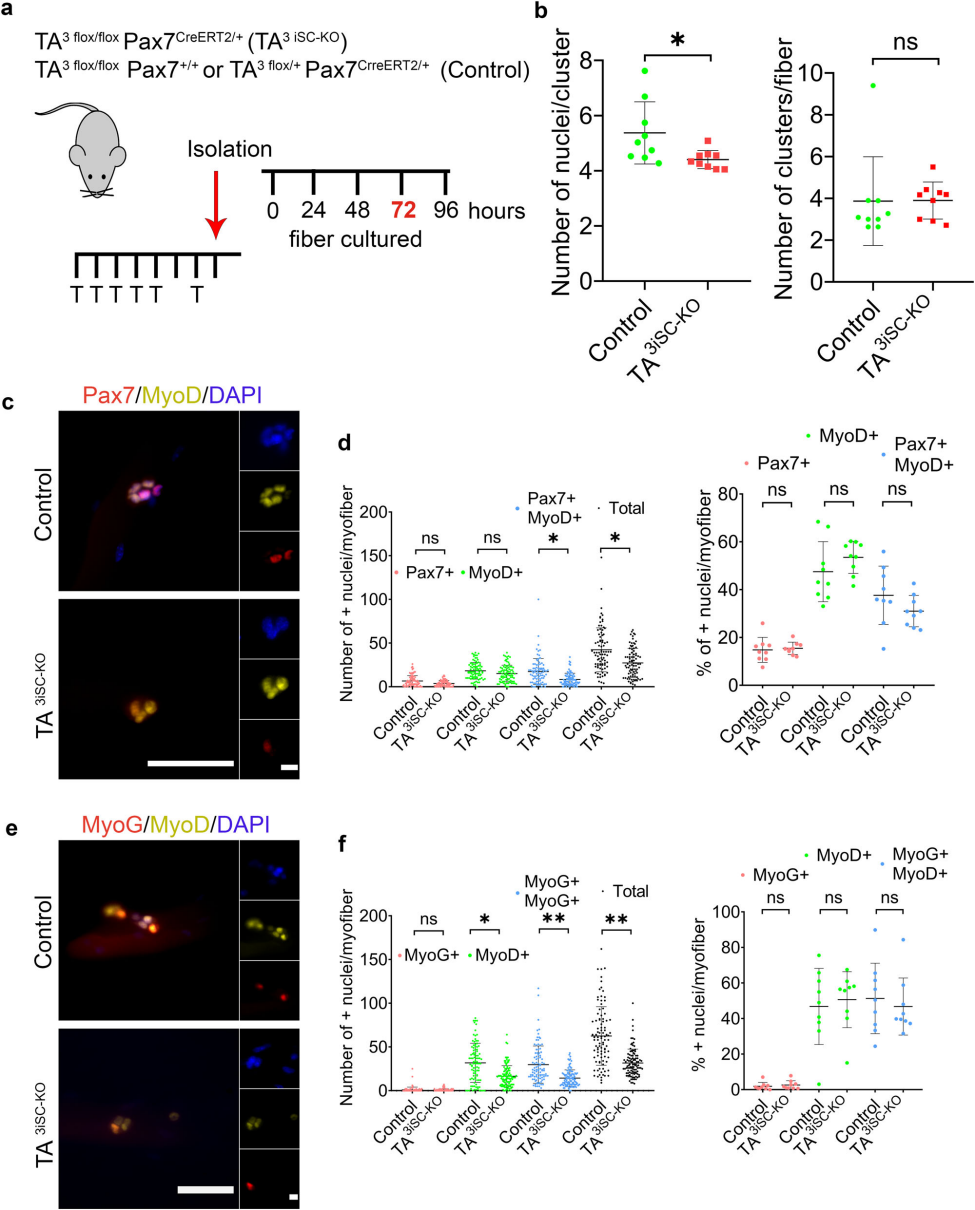
Efficient proliferation of MuSC requires TA^3^. **a** Schematic illustration of tamoxifen injection regime followed by ex vivo culture of myofibers for the times indicated, 72 h shown here. Genotypes of mutant and control mice are shown. **b** Clusters of MuSC were counted and plotted as the total number of nuclei per fibre or the number of clusters per fibre. Fewer MuSC-derived cells were present in TA^3iSC-KO^ compared to controls. **c** Pax7 and MyoD or **e** MyoD and MyoG immunostaining of MuSC progeny on myofibers isolated from control or TA^3iSC-KO^ mice (scale bar = 50 μm). **d** Graphs show the total number of nuclei per myofiber in control and TA^3iSC-KO^ fibres, or the percentage of Pax7^+^ (red), MyoD^+^ (green) or Pax7^+^/MyoD^+^ (blue) double positive cells *p < 0.05, *n* = 9, unpaired, two-tailed *t* test. **f** Graphs show the total number of nuclei per myofiber in control and TA^3iSC-KO^ fibres, or the percentage of MyoG^+^ (red), MyoD^+^ (green) or MyoG^+^/MyoD^+^ (blue) double positive cells *p < 0.05; **p < 0.01, *n* = 9, unpaired, two-tailed *t* test, mean and SD are shown.

To determine cellular defects arising from lack of TA^3^, control and TA^3iSC-KO^ myofibers were cultured ex vivo to characterise activation, proliferation and differentiation of MuSCs in their physiological niche (Fig. 2a). Myofibers were analysed at different time points by immunostaining for Pax7, Myf5, MyoD and MyoG, and nuclei were counted using DAPI. Pax7 expression marks quiescent MuSCs. Once cells are activated, they express Myf5, followed by MyoD and MyoG, which indicates proliferation and differentiation. A proportion of cells maintains or re-expresses Pax7 and downregulates MyoD to self-renew the stem cell pool^46^. At 0, 24 and 48 h, we detected no differences in either the total number of cells or the proportion of cells expressing different markers (Supplementary Figs. 2 and 3 and Supplementary Data 2). However, from 72 h TA^3iSC-KO^ mutant myofibers had fewer MuSC-derived cells compared to controls (Fig. 2b–f and Supplementary Data 1). The number of cell clusters per myofiber was not affected, but there were fewer cells per cluster (Fig. 2b), suggesting a defect in MuSC activation or proliferation. Caspase 3 staining showed no difference between TA^3iSC-KO^ and control myofibers suggesting the reduced cell number was not due to an increase in apoptosis (Supplementary Fig. 4 and Supplementary Data 2). In TA^3iSC-KO^ mutants, the proportion of cells positive for Pax7, or MyoD, or both was not affected, although there were fewer double positive cells (Fig. 2c, d). This was also the case at 96 h (Supplementary Figs. 2 and 3 and Supplementary Data 2). Immunostaining for MyoD and MyoG showed no difference in the proportion of single and double positive cells, indicating that myogenic differentiation was not impaired, although there were fewer cells in total (Fig. 2e, f).

### Loss of TA^3^ in MuSC impairs skeletal muscle repair after injury

**Fig. 3:**
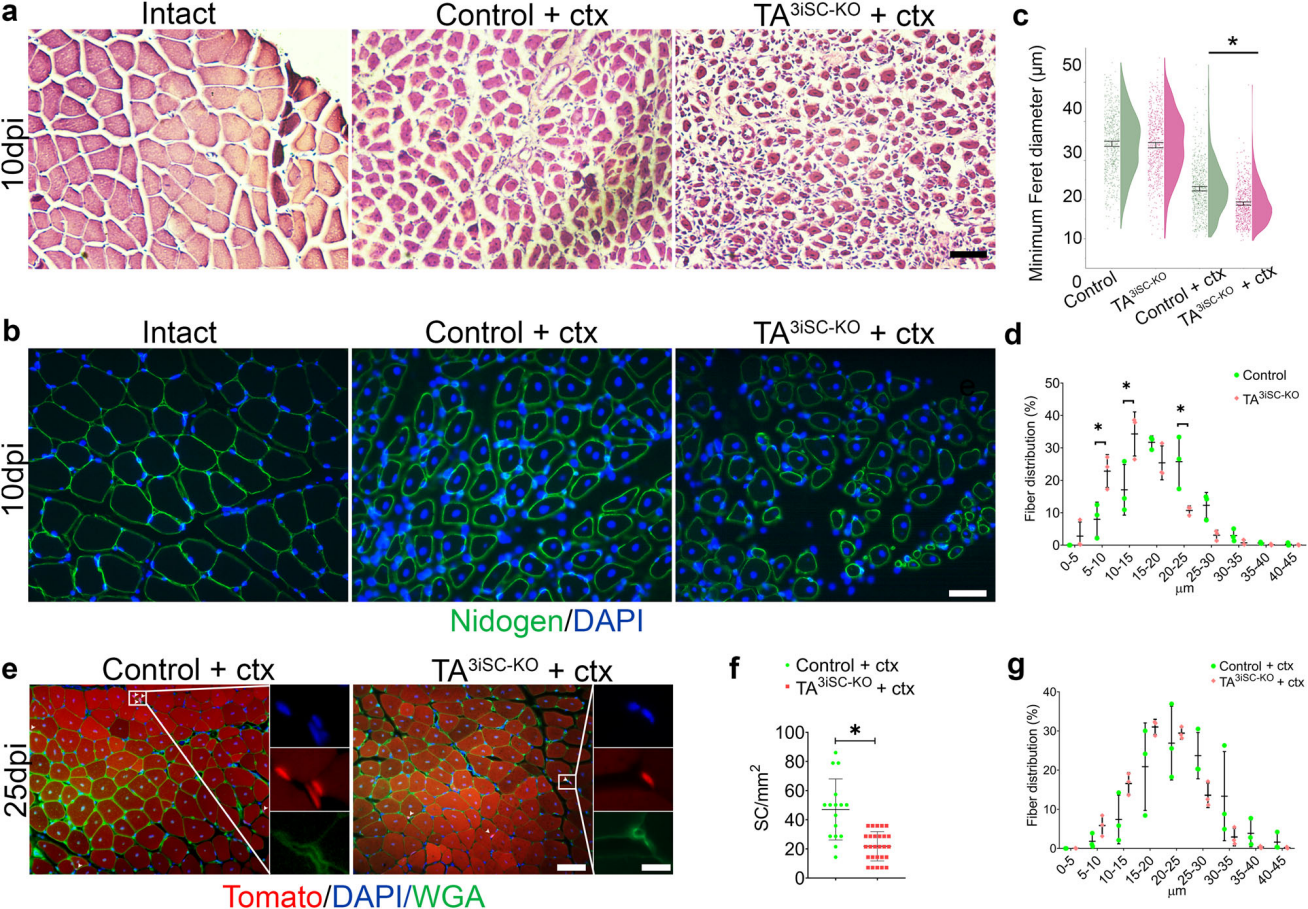
Regeneration of myofibers is impaired after deletion of TA^3^ in MuSCs. **a** Histology images of *tibialis anterior* (TA) muscle transverse sections stained with haematoxylin and eosin. **b** Representative images of TA transverse sections, nidogen staining visualises basement membrane (green), DAPI stains nuclei (blue). Sections from non-injured (intact), and injured control and TA^3iSC-KO^ muscles are shown as indicated, 10dpi = 10 days post-injury. **c** Raincloud plots show size distribution of myofiber minimal Feret diameters in control (green) and TA^3iSC-KO^ (red) mice, non-injured or after cardiotoxin (ctx) injury, *p < 0.05, 95% CI (confidence interval) of the mean is shown. **d** Distribution of minimal Feret diameters of myofibers in control (green) and TA^3iSC-KO^ (red) *tibialis anterior* muscles after 10dpi. **e** Histology of control and TA^3iSC-KO^ transverse muscle sections at 25dpi, red shows Td-Tomato, green shows the basement membrane (BM) stained with wheat germ agglutinin (WGA), DAPI nuclei in blue. White arrowheads show MuSC identified based on Td-Tomato and their location beneath the BM. **f** At 25dpi fewer MuSC were counted in the mutant. **g** Distribution of minimal Feret diameters of myofibers in control (green) and TA^3iSC-KO^ (red) *tibialis anterior* muscles after 25dpi. Scale bars = 50 µm, all conditions *n* = 3 mice per genotype, *p < 0.05, unpaired, two-tailed *t* test, mean and SD are shown.

Next, we determined the effect of primary cilia loss on the ability of skeletal muscle to repair in vivo, following injury caused by cardiotoxin (ctx) injection. Deletion of TA^3^ was induced in MuSC prior to injury and hindlimb muscles were dissected on day 5, 10 or 15 post-injury. Histology of transverse cryosections and nidogen staining of basement membranes showed disorganised myofibers at day 10 post-injury (Fig. 3a, b). DAPI staining confirmed centrally located myonuclei in regenerated myofibers compared to non-injured control muscles, where myonuclei are found in the periphery (Fig. 3b). In uninjured muscles, the distribution of Feret diameters was identical in control and TA^3iSC-KO^ myofibers (Fig. 3c). However, following injury regenerating TA^3iSC-KO^ myofibers had smaller average Feret diameters compared to controls (Fig. 3c, d and Supplementary Data 1).

To determine whether TA^3iSC-KO^ mice would eventually regenerate muscle more completely, we also examined Feret diameters at 25 days post-injury. The average Feret diameters were similar when control muscles had reached normal fibre size (Fig. 3e, g and Supplementary Data 1). This shows muscle regeneration is delayed in absence of TA^3^. Furthermore, the number of MuSC positive for Pax7 and Td-Tomato was reduced 25 days post-injury (Fig. 3e, f and Supplementary Data 1). This suggests an effect on MuSC self-renewal in TA^3iSC-KO^ mice compared to controls.

### Regeneration is more severely compromised in TA^3iSC-KO^ after repeat injury

**Fig. 4:**
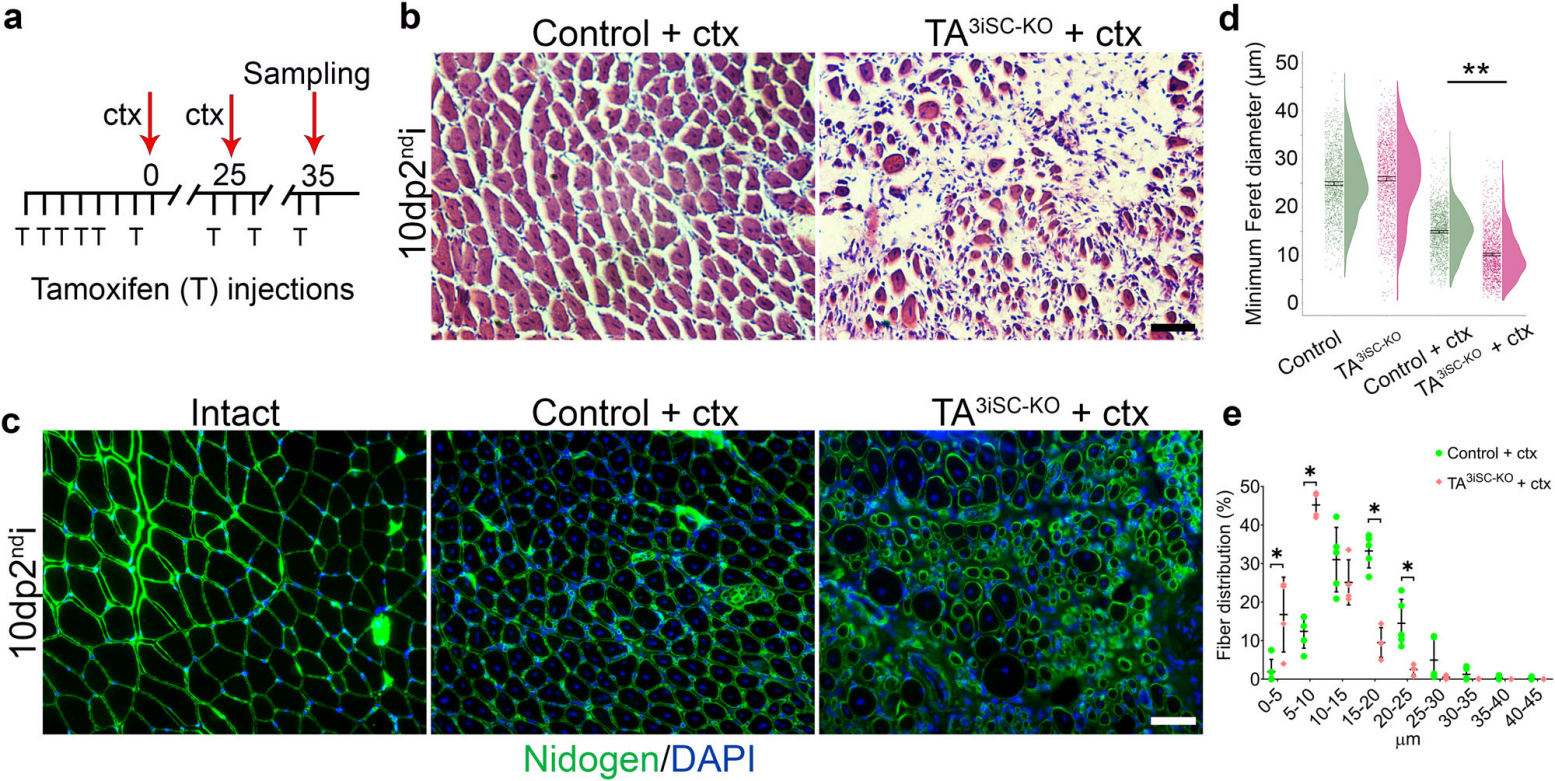
A second injury enhances regeneration defects in TA^3iSC-KO^ mice. **a** Schematic of the tamoxifen (T) and ctx injection regime. The first injury on day eight was followed by a second cdx injection 25 days later. Analysis was performed 10 days post-second injury (10dp2^nd^i). **b** Histology shows good recovery in controls but poor recovery in TA^3iSC-KO^ mice. **c** Transverse sections stained with nidogen (green) and DAPI (blue) at 10dp2^nd^i. Sections from non-injured (intact) and injured muscles from control and TA^3iSC-KO^ mice are shown as indicated. **d** Raincloud plots show the size distribution of myofiber minimal Feret diameters in control (green) and TA^3iSC-KO^ (red) mice, either non-injured or 10 days after the second injury (10dp2^nd^i) (ctx). **e** Distribution of minimal Feret diameters in control (green) and TA^3iSC-KO^ (red) *tibialis anterior* muscles after repeat injury. Scale bars = 50 µm, *n* ≥ 3 mice per genotype, *p < 0.05, **p < 0.01, unpaired, two-tailed *t* test, mean and SD, or 95% CI (confidence interval) of the mean are shown.

We used repeat injury to confirm that self-renewal of MuSCs was affected. A second injury was applied after a 25-day recovery period (Fig. 4a) and muscle was examined on day 10 after the second injury. In control mice, histology was similar 10 days after repeat injury compared to single injury. However, TA^3iSC-KO^ mutants had more severe fibrosis after repeat injury compared to single injury (Fig. 4b). Furthermore, myofiber diameters were smaller in TA^3iSC-KO^ mutant compared to control muscles 10 days post repeat injury (Fig. 4d, e and Supplementary Data 1), and myofiber diameters were more reduced after repeat injury compared to single injury (~10 µm compared to ~18 µm) (Figs. 3c and 4d). This confirms that MuSC are depleted after a first injury and is consistent with the notion that self-renewal is affected. In non-injured control and mutant muscle the fibre diameters were similar.

### Single cell analysis reveals deregulated signalling pathways in TA^3iSC-KO^ MuSCs

To determine the mechanisms underlying the loss of MuSC after 72 h in ex vivo cultures and the impaired muscle regeneration after injury, we established molecular profiles of MuSC and their progeny. We first examined whether there are immediate effects on gene expression after TA^3^ deletion and performed differential transcriptomics of quiescent MuSC isolated from control and TA^3iSC-KO^ mice. Pax7-Zs-green fluorescence^42^ was used to FACS-isolate between 30,000 and 50,000 MuSCs from dissected hindlimb muscles, immediately following tamoxifen-induced TA^3^ deletion, for RNAseq. Principle component analysis did not clearly separate the control and TA^3iSC-KO^ samples and only few differentially expressed genes were identified, with 35 genes up-regulated and 27 genes down-regulated. GO-term analysis did not highlight specific pathways or processes that were enriched. Deletion of TA^3^ did not lead to significant molecular differences in quiescent MuSCs, however, we noticed that several genes associated with ECM were differentially expressed, such as TIMP1, LAMB1 and ITGA8 (Supplementary Fig. 5).

**Fig. 5:**
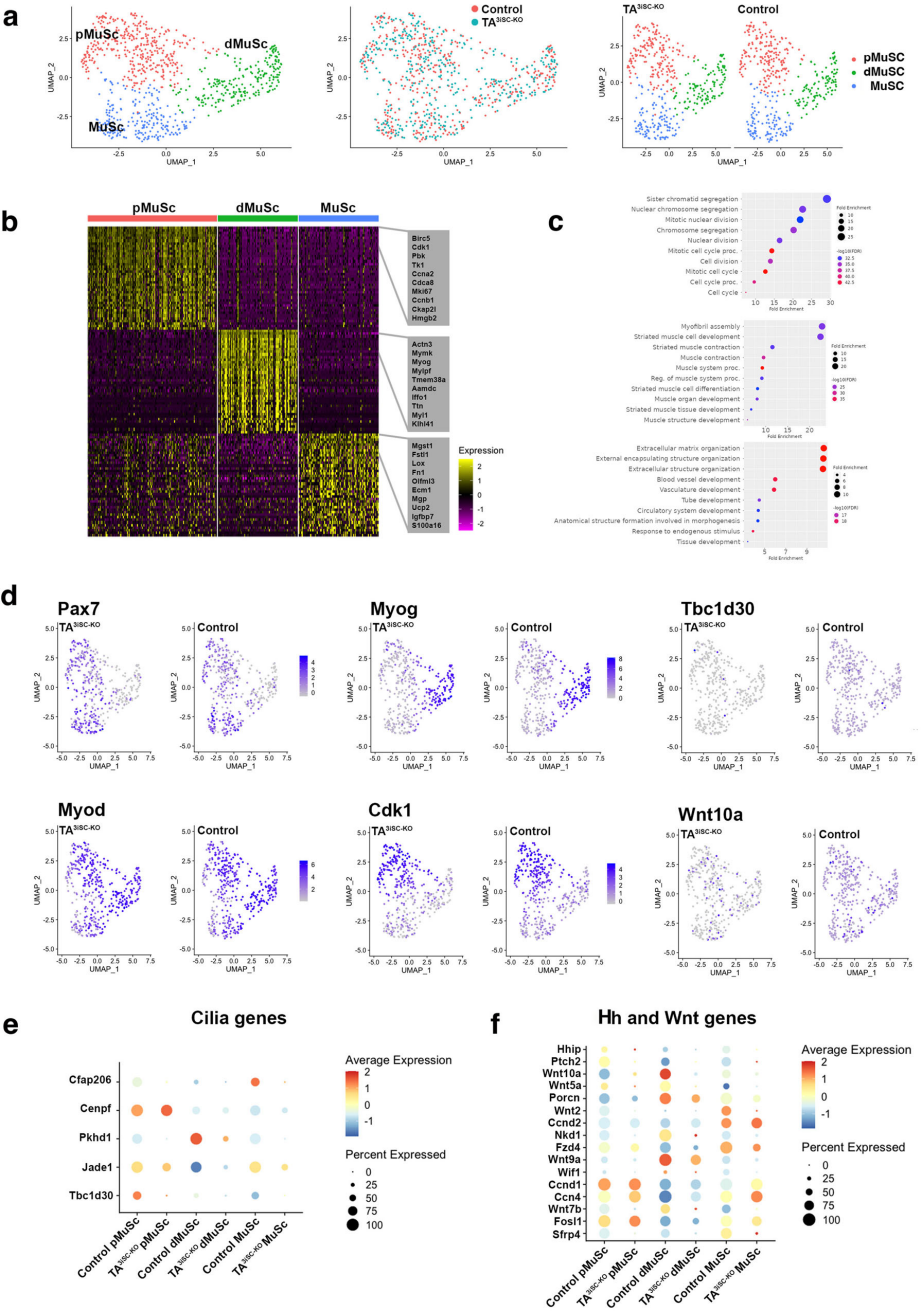
Loss of TA^3^ leads to deregulation of signalling pathways in MuSC progeny. **a** UMAP clustering of Td-Tomato MuSC progeny isolated from ex vivo cultured myofibers after 72 h identifies equal proportions of proliferating MuSC (pMuSC), differentiating MuSC progeny (dMuSC) and MuSC populations in control and TA^3iSC-KO^ myofibers. **b** Heatmap shows the top 40 most variably expressed genes between the three MuSC clusters, with the top 10 genes highlighted in grey. **c** Gene Ontology (GO) functional terms enriched in pMuSC (top), dMuSC (middle) and MuSC (bottom) populations are shown. **d** Expression of different marker genes across the three clusters in control and TA^3iSC-KO^ MuSC. Markers shown are Pax7, MyoD, MyoG, Cdk1, Tbc1d30 and Wnt10a. **e** Comparison of the expression of cilia genes and **f** Hedgehog (Hh) and Wnt signalling components across each of the three clusters.

To determine the effects of TA^3^-loss on MuSC and their progeny as they go through the regenerative cycle, we performed single cell transcriptomics. Myofibers were cultured ex vivo as before. After 72 h, Td-Tomato fluorescent MuSCs were isolated by FACS, sorted into plates and sequenced using Smart-Seq2. From three independent biological replicate samples each, for control and TA^3iSC-KO^ MuSC, we retained 832 MuSC after quality control. Downstream scRNA-seq analysis was performed using Seurat^47^. The cells grouped into three clusters based on distinct transcriptional programs and expression of relevant marker genes (Fig. 5a, b). The top 40 genes identified as predominantly expressed in these three clusters are shown (Fig. 5b). A population of cells characterised by Pax7 was separated further into two distinct clusters (Fig. 5a, d). GO-terms related to cell proliferation were enriched in one of these Pax7 positive clusters (Fig. 5c) and the top 10 differentially expressed genes included cyclin-dependent kinase 1 (Cdk1) (Fig. 5b, d). This cluster was designated proliferating, pMuSC. The second cluster comprising Pax7 expressing cells was designated MuSC. Enriched GO-terms included extracellular matrix (ECM) organisation (Fig. 5c), Fibronectin (Fn1) and Extracellular matrix protein 1 (Ecm1) were among the top 10 differentially expressed genes. The third cluster, dMuSC, consisted of differentiating cells characterised by MyoG expression. GO-terms were enriched for genes associated with myofibril assembly, striated muscle development and contraction (Fig. 5c). The top 10 differentially expressed genes include myogenin (MyoG), Actinin alpha3 (Actn3) and titin (Ttn). In both control and TA^3iSC-KO^, the different populations of MuSC progeny are distributed similarly across all clusters (pMuSC: control = 188, TA^3iSC-KO^ = 183; dMuSC: control = 112; TA^3iSC-KO^ = 119; MuSC: control = 115; TA^3iSC-KO^ = 115) (Fig. 5a). This is consistent with our analysis of ex vivo cultured myofibers by immunostaining, where we detected no change in the proportion of cells labelled by Pax7, Myf5 or MyoG (Fig. 2).

Despite a similar distribution of cells across each cluster, differential analysis revealed changes in gene expression between control and TA^3iSC-KO^. The expression of cilia associated genes was reduced in TA^3iSC-KO^ MuSC. This included Cilia and Flagella Associated Protein 206 (Cfap206) and TBC1 Domain family member 30 (Tbc1d30), a protein involved in regulation of cilia assembly (Fig. 5d, e). Examination of signalling pathways showed that genes involved in Hh signalling were reduced in MuSC lacking TA^3^, including Hedgehog interacting protein (Hhip) and the Patched 2 receptor (Ptch2) (Fig. 5f). Furthermore, expression of many Wnt ligands was reduced in TA^3iSC-KO^ either across all three clusters (Wnt10a, Wnt5a, Wnt7b), or specifically in the Pax7 positive clusters, pMuSCs and MuSCs (Wnt2, Wnt9a) (Fig. 5d, f). We also noticed that expression of both positive and negative regulators of Wnt signalling was reduced in TA^3iSC-KO^ MuSC progeny. Differentially expressed genes include porcupine (Porcn), which mediates Wnt palmitoylation required for activity, naked cuticle homologue 1 (Nkd1) and secreted frizzled related protein 4 (Sfrp4), which inhibit Wnt signalling. Because components of Hh and Wnt signalling were deregulated in TA^3iSC-KO^ cells, we next tested whether pharmacological activation of these pathways could rescue muscle repair after injury.

### Pharmacological inhibition of GSK3β-kinase restores muscle regeneration in TA^3iSC-KO^ mice

**Fig. 6:**
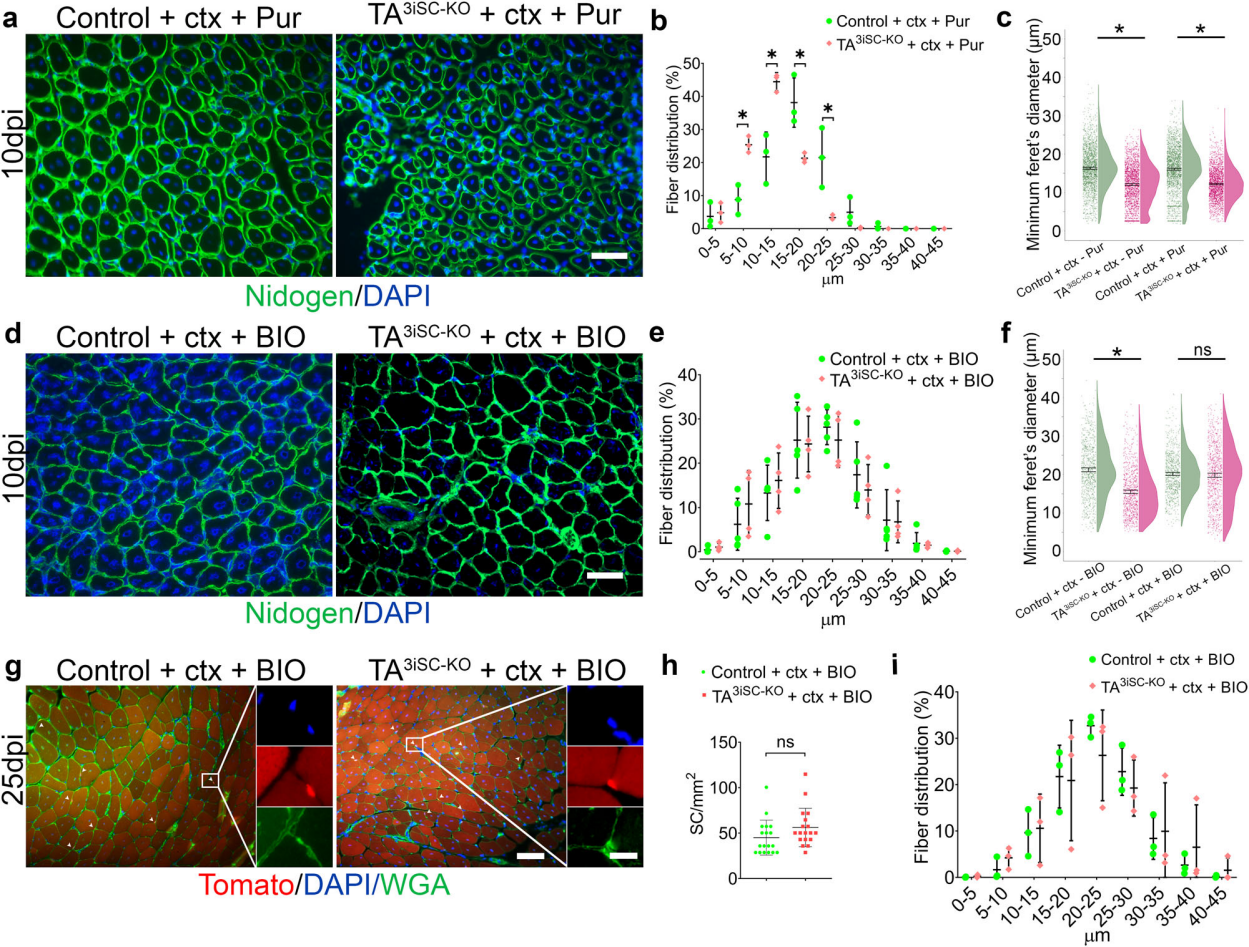
Inhibition of GSK3β by BIO restores muscle regeneration. **a**, **d** Transverse sections 10 days post-injury (10dpi) stained for nidogen (green) and DAPI (blue). Sections are shown for control and TA^3iSC-KO^ muscles, **a** co-injected with ctx and purmorphamine (Pur), or (**d**) co-injected with ctx and BIO (scale bar = 50 µm). **b**, **e** Histograms and **c**, **f** raincloud plots show the range of myofiber diameters in control (green) or TA^3iSC-KO^ (red) mice, injected with ctx and Pur (**b**, **c**) or with ctx and BIO (**e**, **f**). **g** Histology of control and TA^3iSC-KO^ transverse muscle sections at 25dpi, red shows Td-Tomato, green shows the basement membrane (BM) stained with wheat germ agglutinin (WGA), DAPI nuclei in blue. White arrowheads show MuSC identified based on Td-Tomato and their location beneath the BM. **h** At 25dpi similar numbers of MuSC were counted in control and mutant in presence of BIO. **i** Distribution of the range of myofiber diameters in control (green) or TA^3iSC-KO^ (red) mice, injected with ctx and BIO at 25dpi. *p ≤ 0.05 calculated by unpaired, two-tailed *t* test, *n* ≥ 3 mice, mean and SD, or 95% CI (confidence interval) of the mean are shown.

The primary cilium is a nexus for Hh signalling and it has been shown that the co-receptor, Smoothened (Smo), which regulates downstream processing of Gli proteins to generate transcriptional activators, exhibits signalling competency in the absence of ciliary accumulation^48^. Thus, we used purmorphamine (Pur), a pharmacological activator of Smo, to determine whether this could restore the regenerative defect in TA^3iSC-KO^ mice. We found, however, that after co-injection of Pur with ctx myofiber diameters remained reduced on day 10 in TA^3iSC-KO^ muscle compared to controls (Fig. 6a–c and Supplementary Data 1). Thus, treatment was not sufficient to restore muscle regeneration. We examined expression of Hh responsive genes to assess whether TA^3iSC-KO^ MuSC can activate Hh signalling in response to Pur. We found that Gli1 and Ptch1 expression was increased in MuSC isolated by FACS (Supplementary Fig. 6 and Supplementary Data 2).

GSK3β kinase is a major negative regulator of β-catenin dependent (canonical) Wnt signalling. It is highly expressed in muscle and inhibition of GSK3β enhances self-renewal of embryonic stem cells^43^. Given that expression of Wnt ligands was reduced in TA^3iSC-KO^ MuSC (Fig. 5f), we examined the effect of GSK3β inhibition. Muscle regeneration was restored after co-injection of ctx with BIO (6-bromoindirubin-3′-oxime) and myofiber diameters were similar in TA^3iSC-KO^ and control mice, at day 10 and 25 post-injury (Fig. 6d–f, i and Supplementary Data 1). This indicates that BIO could ameliorate the regeneration defect in absence of primary cilia. Since BIO-mediated GSK3β inhibition activates β-catenin dependent Wnt signalling and Hh signalling, we assessed expression of Axin2, a Wnt target gene, alongside Gli1 and Ptch1. In FACS isolated MuSC, application of BIO induced expression of Axin2 and to a lesser extent the expression of Gli1 and Ptch1 (Supplementary Fig. 6 and Supplementary Data 2). This suggests that MuSC activate predominantly β-catenin dependent Wnt signalling in response to BIO.

To determine whether BIO treatment restored the number of MuSC, we counted Td-Tomato positive cells in vivo on day 25 after injury (Fig. 6g, h). In presence of BIO, TA^3iSC-KO^ muscles had similar numbers of MuSC compared to controls following ctx-induced injury. Contrary to previous observations, this suggests that—in absence of primary cilia—canonical Wnt activation promotes muscle regeneration by restoring MuSC self-renewal.

## Discussion

Quiescent muscle stem cells have a primary cilium, which is dynamically regulated during the myogenic cycle and is reassembled in self-renewing stem cells^10,35,36,49^. Myofibers isolated from aged muscles have fewer MuSCs with a primary cilium, indicating that cilia might be lost with age or become less stable^37^. Ciliopathies, a group of genetic disorders resulting from perturbation of ciliary proteins in human, affect several different tissues including muscle. For example, Joubert Syndrome, which can result from mutations in the Talpid3 gene (KIAA0586)^13^, is associated with hypotonia^50,51^. However, the role of primary cilia in skeletal muscle is not fully understood. Here, we use inducible deletion of an essential ciliary protein, TA^3^, in MuSC to show that primary cilia are critical for efficient regeneration after muscle injury in vivo. Using single cell sequencing of MuSC, we found that both Hh and Wnt signalling were deregulated in absence of TA^3^. Importantly, we discovered that pharmacological activation of Wnt signalling, but not Hh signalling, can restore muscle repair in the absence of a primary cilium. In particular BIO-mediated inhibition of GSK3β rescued the impairment in regeneration and restored the number of MuSC, which were reduced after loss of TA^3^ and cilia disruption. These findings provide insights into potential avenues for treatments in muscle disease or ageing.

It has previously been proposed that the primary cilium may provide a potential target for regenerative therapies^37,39,52^ and recent work has implicated the cilium in muscle regeneration. Specifically, deletion of the intra-flagellar transport protein, IFT88, in MuSCs led to a shift toward myofibers with reduced cross-sectional area post-injury^37^. This agrees with our finding that loss of primary cilia, after conditional deletion of another ciliary protein, TA^3^, in MuSC impairs muscle repair and that, following injury, regenerating TA^3iSC-KO^ myofibers had smaller diameters compared to controls. In addition, we found that the number of Pax7/Td-Tomato positive MuSC were reduced and that repeat injury enhanced the regeneration defect and led to a more severe phenotype (Fig. 4). This suggests, MuSC are depleted and their expansion and self-renewal is affected in absence of TA^3^.

It is striking that MuSC lacking GLI3, which acts predominantly as a repressor, display rapid cell-cycle entry, increased proliferation and expansion of the stem cell pool^39^, whereas loss of cilia inhibits proliferation and MuSC expansion. The latter was observed after deletion of IFT88 in MuSCs^37^ and in this report where, following TA^3^ deletion, the number of MuSC progeny was reduced on ex vivo cultured myofibers (Fig. 2) and in vivo after completion of the regenerative cycle (day 25 post-injury) (Fig. 3e, f). The opposite effects observed are likely due to the dichotomy in the Hh pathway, with the GLI proteins having either activator or repressor function. This suggests the cilium is necessary to maintain an appropriate balance of GLI activator and repressor proteins^38^.

Molecular profiling of individual MuSCs showed that expression of cilia related genes was significantly reduced in cells lacking TA^3^ (Fig. 5e). UMAP clustering identified a population of MuSCs positive for Pax7. This population could be sub-divided into two clusters, one of which was characterised by the expression of genes related to cell division, such as Cdk1 (Fig. 5d) and comprised mitotically active, proliferating cells. The second cluster of MuSCs positive for Pax7 was enriched for genes associated with ECM related processes. We propose that this cluster comprises MuSCs that are close to quiescence, similar to those recently described after single cell sequencing of whole hindlimb muscle^53^. This cluster may also include self-renewed MuSCs that have entered quiescence, as some cells express low transcript levels for myogenic regulatory factors, such as MyoD (Fig. 5e) or Myf5.

During embryogenesis, Sonic hedgehog signalling regulates basement membrane components, such as laminin-111^54–56^. This is crucial for myotome formation and basement membranes are disrupted in the TA^3^ mutant^13^. It is therefore interesting to note that TA^3^ deletion led to differences in expression of ECM related genes in quiescent MuSC isolated immediately following the deletion of TA^3^. This included tissue inhibitor or matrix metalloprotease 1 (TIMP1), matrillin 2 (Matn2), laminin subunit beta 1 (LAMB1), integrin alpha 8 (Itga8) and ADAM Metallopeptidase Domain 12 (ADAM12), which were all upregulated in absence of TA^3^. However overall, there were few significant differences in MuSC transcriptomes at this time point (Supplementary Fig. 5). This is not unexpected as these cells are transcriptionally not very active. The differences we do see may also be due to partial activation induced by the isolation process^57–60^.

As primary cilia are implicated in both Hh and Wnt signalling, we examined the expression of components of these pathways in control and TA^3iSC-KO^ MuSCs using scRNA-sequencing. Differential analysis showed that both pathways were deregulated in absence of TA^3^ (Fig. 5f). During regeneration, canonical Wnt signalling is activated and has been implicated in regulating myogenic lineage progression^61^. However, β-catenin dependent Wnt signalling is only transiently active in expanding myoblasts, and its restriction is crucial for successful muscle regeneration^28–30^. Prevailing evidence suggests that Wnt signalling is particularly involved in the regulation of satellite cell differentiation and self-renewal^26^. For example, Wnt activates follistatin and myoblast fusion during terminal differentiation^62^. Myoblasts lacking β-catenin show delayed differentiation, whereas constitutively active β-catenin promotes precocious differentiation^30^. During embryonic development, Wnt signalling is also important for myogenesis^63–65^. Furthermore, Sonic hedgehog synergises with Wnt to activate myogenesis in paraxial mesoderm progenitors in both mouse and chick^16–18,66^. Genetic experiments targeting Smo or Gli transcriptional effectors, show that Shh acts cell-autonomously in limb muscle progenitor cells by initiating Myf5 expression and regulating directional muscle cell migration in the distal limb^21,22^. Thus, these signalling pathways are important in embryonic and adult myogenesis with complex and context dependent functions. Our findings indicate that modulation of signalling pathway activity by the primary cilium adds another facet to the complex regulation of regenerative myogenesis.

It was particularly striking that Hedgehog interacting protein (Hhip), Patched2 (Ptch2) and several Wnt ligands, Wnt10a, Wnt5a, Wnt9a, Wnt2 and Wnt7b, were significantly reduced in TA^3iSC-KO^ MuSCs (Fig. 5f). This observation prompted us to attempt pharmacological rescue of the regeneration defect. Co-injection of ctx and purmorphamine (Pur), a smoothened agonist^44^, activated Hh target genes in MuSCs, however did not restore muscle repair (Fig. 6a-c, j). This agrees with a previous report, where injection of SAG1.3, another Hh pathway agonist, had no effect in mice with IFT88^−/−^ MuSCs, either on muscle strength or MuSC proliferation^37^. Thus, the Hh mediated effects on MuSC during the regenerative process appear to be completely cilia dependent. In contrast, co-injection of ctx with an inhibitor of GSK3β kinase (BIO) restored muscle repair independently of primary cilia (Fig. 6d–f), this was associated with restored numbers of MuSC (Fig. 6g, h).

GSK3β kinase is highly expressed in muscle; it regulates many signalling pathways including Shh, Wnt and Notch. It has been shown that BIO mediated suppression of GSK-3 activity promotes Wnt/beta-catenin signalling to preserve the pluripotency of human and mouse embryonic stem cells^43^. GLI2, which is part of the Hh pathway, can also be phosphorylated by GSK-3^67,68^, however, BIO did not significantly activate the expression of Hh target genes in MuSCs (Fig. 6j). Instead, we found that in response to BIO MuSC activated expression of Axin2, a target gene of canonical Wnt signalling. However, Axin2 is also a repressor of the pathway as it inhibits β-catenin. As mentioned above, the regulation of Wnt activity levels is crucial to maintain proliferation and to control timely myogenic differentiation^28–30^. We hypothesise that co-injection of BIO achieved appropriate levels of Wnt activity as the regenerative response was restored in absence of cilia, both myofiber cross-sectional area and MuSC numbers were comparable to controls.

GSK-3β can also regulate the stability of surface Notch receptors and there is extensive cross-talk between Wnt and Notch signalling^69,70^. However, our transcriptomics analysis did not identify differentially expressed Notch pathway components.

In summary, we show that BIO restores muscle regeneration and that this works predominantly through activation of Wnt signalling, which can overcome the lack of TA^3^ and primary cilia. Thus, our study identifies a therapeutic target in MuSCs that could be of interest in muscle degenerative disease or in ageing. However, more work is needed to explore cell autonomous and cell non-autonomous effects of the treatment.

## Methods

### Animal models

All mice were housed in the Disease Modelling Unit (DMU) of the University of East Anglia. Experimental procedures were performed in accordance with the Animal (Scientific Procedures) Act 1986 (ASPA) under the UK Home Office Project Licences 70/8824 and PP3253888. All lines were maintained on a C57BL/6J background: Pax7^CreERT2/+^^41^, Talpid3^flox/flox^^15^. B6.Cg-Tg(Pax7-ZsGreen)^1Kyba/J^ (kindly provided by M. Kyba, University of Minnesota, USA^42^; and Rosa26-td-Tomato (Ai14, The Jackson Laboratory). Mice carrying the TA^3^ floxed allele were crossed with mice expressing the tamoxifen inducible cre-recombinase, CreERT2, under the control of the endogenous Pax7 promoter (Pax7^CreERT2^), generating control mice and TA^3 icSC-KO^ in the same litter. Experimental mice were 8–12 weeks old, males and females were used. For genotyping DNA was extracted from ear notches and polymerase chain reaction (PCR) was performed as described^15,41^.

### IP injections

Tamoxifen was injected for 5 consecutive days (3 mg/40 g body weight), followed by a rest day and another tamoxifen injection on day 7. During the regeneration phase, tamoxifen was administered every other day.

### Muscle injury model

Mice were anaesthetised with isofluorane and muscle injury was induced with 50 µl of a 10 µM cardiotoxin (ctx) (Latoxan) solution in the right *tibialis anterior* muscle. The un-injured left leg served as control. Muscles were isolated at the indicated time points after injury. For double injury experiments, a second injury was induced after 25 days of recovery from the first injury. Muscle was then analysed 10 days post repeat-injury. For the rescue experiments, purmorphamine (2 µM) or BIO (10 µM) were injected together with ctx.

### Muscle fibre isolation

Following administration of tamoxifen *extensor digitorum longus* (EDL) muscles were isolated from 8- to 12-week-old mice and incubated in Collagenase I (2 mg/ml, Worthington) for 90 min at 37 °C, after which muscles were transferred to BSA (Sigma) coated dishes containing DMEM (Gibco) and PenStrep (Gibco). EDL muscles were subjected to repeated trituration through descending diameter glass Pasteur pipettes until single myofibers separated from the isolated muscle. Single myofibers were selected and cultured in 10% Horse Serum (HS), 1% Chick Embryo Extract (CEE) and PenStrep in DMEM.

### Histology and immunofluorescence

To confirm the degree of injury and regeneration, at the given time points mice were sacrificed and skeletal muscles were harvested, fixed in 4% PFA, embedded in OCT after sucrose treatment and transverse cryosectioned (10 µm), followed by haematoxylin–eosin.

For immunostaining transverse section were blocked with 5% Normal Goat Serum in Tris-buffered saline containing 0.1% Tween 20 (TBS-T), followed by incubation with Nidogen1^71^ after washing Alexa Fluor 488 secondary antibody and DAPI (0.5 µg/ml) were applied. Slides were mounted with gelvatol for imaging. Anti-wheat germ agglutinin (WGA) conjugated with Alexa Fluor 488 (Invitrogen W11261) was incubate for 15 min with a 1:200 dilution.

Muscle fibres were were fixed for 10’ with prewarmed 2% Paraformaldehyde (PFA) at indicated time points, washed with PBS and permeabilised by adding 0.5% TritonX100 and blocked for 2 h with 10% NGS in TBS-T at 37 °C. Primary antibodies against Pax7 (DSHB), Myf5, MyoD (Santa Cruz) and Myogenin (DSHB) were used at a 1:50 dilution in 5% NGS/TBS-T and incubated at 4 °C overnight. Samples were extensively washed with TBS-T before secondary antibodies (Jacksonimmuno) were applied together with DAPI for 2 h in the dark. Fibres were washed in TBS-T and TBS, and 10-20 fibres were mounted in gelvatol on a glass slide for imaging.

For immunostaining of primary cilia fixed fibres were permeabilised with 0.5% TritonX100 and 0.5% Nonidet P40 and 2% MeOH. Primary antibodies used were directed against Arl13B (Proteintech, 1:300) and Pax7 (DSHB; 1:7.5). Secondary antibodies used were goat anti-mouse IgG-Fab2 488 and goat anti-rabbit Alexa fluor 546 (1:375) (Jacksonimmuno).

### Microscopy and image analysis

Fluorescence images of muscle sections and muscle fibres were taken using a Zeiss AxioPlan 2ie microscope. Images of muscle sections were processed in Fiji (ImageJ) after being pixel corrected in Ilastik and the minimum Feret diameter was measured.

### RNA extraction and real-time qPCR

RNA from the Td-Tomato positive cells was extracted using trizol and qPCR was performed in triplicates by using the RNA to CT-1-step Sybr green kit (Life Technologies Ltd). Primers to detect transcripts of Hh and Wnt targets are in Supplementary Table 1.

### Fluorescence-activated cell sorting

Quiescent MuSCs (Pax7-ZsGreen+) were obtained from uninjured hindlimb muscles. For scRNAseq cells were isolated from ex vivo cultured myofibers (72 h) and Td-Tomato positive MuSCs were sorted using the BD FACSMelody cell sorter (BD Biosciences, San Jose, California) directly into lysis buffer in 96-well plates. From three independent experiments we recovered two plates each from control and TA^3iSC-KO^ myofibres, generating 6 plates per genotype.

### Single-cell RNA-seq

Amplified cDNA was generated from sorted cells using the SmartSeq2 protocol^72^. Sequencing libraries were generated using an automated, reduced-volume version of the Nextera XT protocol (Illumina) using the SPT Labtech Mosquito LV. Libraries were pooled (384-plex) and sequenced on a NovaSeq 6000 (Illumina, San Diego) in paired end, dual index mode. Raw Illumina sequencing data were analysed to obtain a single-cell expression matrix object. Subsequent analysis was performed in R using Seurat version 3^47^. Cells showing gene counts lower than 1000 and a mitochondrial gene expression percentage higher than 5% were excluded from further analysis. Within Seurat, data were normalised using NormalizeData (normalization.method = LogNormalize, scale.factor = 10000) and data from multiple samples were merged using the FindIntegrationAnchors and IntegrateData commands.

### Statistics and reproducibility

A minimum of three independent experiments (or animals) was used for all assays. Statistical analysis was carried out using Graphpad Prism software. Multiple data points were collected per mouse to generate the population mean and represent it as raincloud plots using ggplot2 in R-studio. These values were used to compare between control and mutant mice. Unpaired, two tailed Student’s *t* tests were used to calculate statistical significances, shown as *p* values, means and SD are shown. Plots were generated using Graphpad Prism and comparison was done at each fibre range.

### Reporting summary

Further information on research design is available in the Nature Portfolio Reporting Summary linked to this article.

### Supplementary information

Supplementary Information

Description of Additional Supplementary Files

Supplementary Data 1

Supplementary Data 2

Reporting Summary

## Data Availability

For bulk and single-cell transcriptomics, the raw sequencing data can be accessed on the NCBI-SRA archive under accession number BioProject PRJNA981098. Other raw data, cell counts and myofiber diameters, are available as Supplementary Data files for the main (Supplementary Data 1) and Supplementary Figures (Supplementary Data 2).
