# Supplementary material for: Absence of the primary cilia formation gene Talpid3 impairs muscle stem cell function
Source: Commun Biol. 2023 Nov 4;6:1121. doi: 10.1038/s42003-023-05503-9 (PMC10625638; doi:10.1038/s42003-023-05503-9)
Supplement: Supplementary file 1 — Supplementary Information [file 42003_2023_5503_MOESM1_ESM.docx]

**Supplementary Figures, Figure legends and Tables**

**
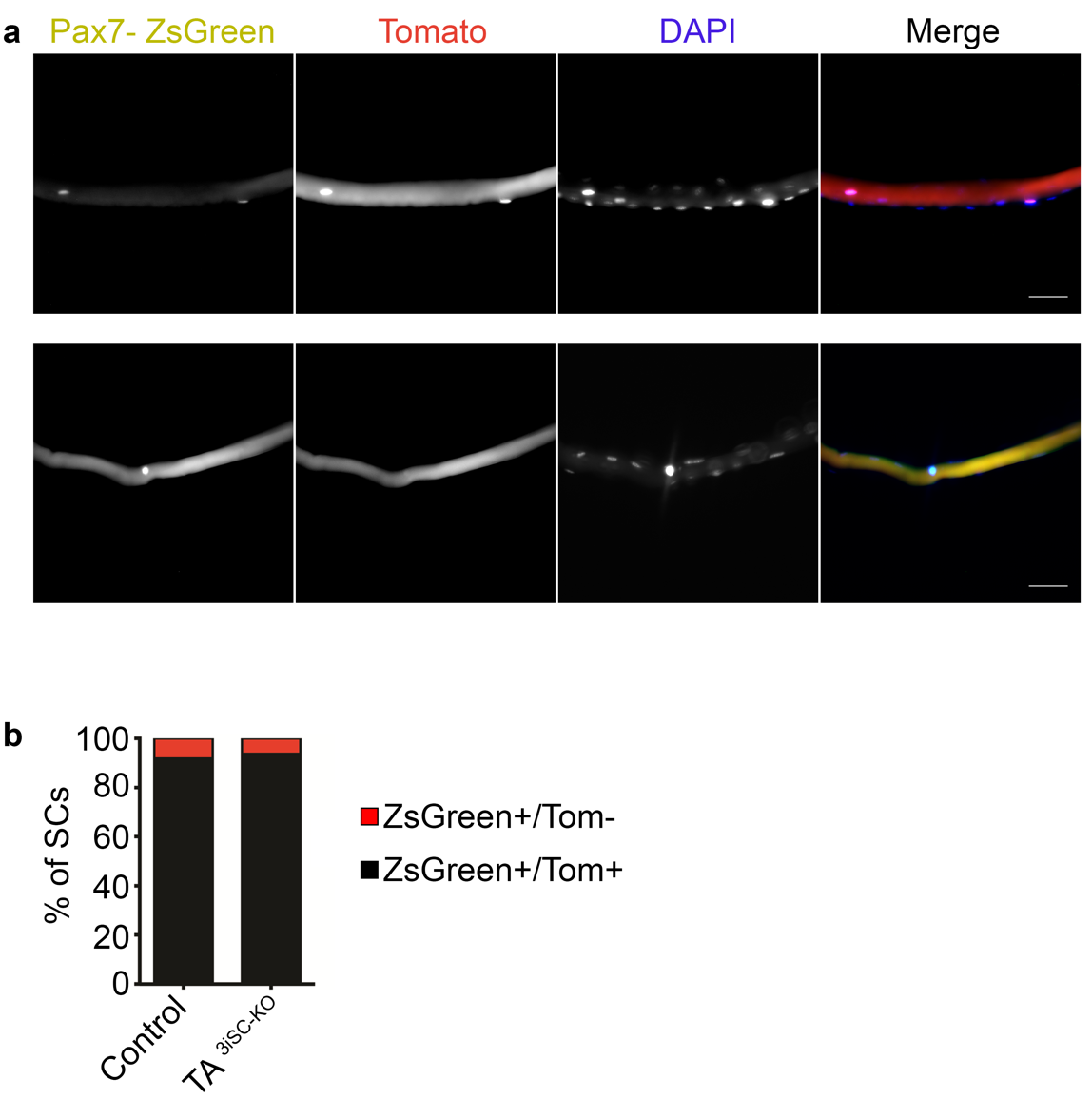
**

**Supplementary Figure 1: Three tamoxifen injections achieve a high recombination efficiency. (a)** Images show single myofibers isolated from control or TA^3iSC-KO^ mice. Individual channels show MuSC that are positive for Pax7-ZsGreen, or Td-Tomato. DAPI staining reveals MuSC nuclei and myonuclei. The merged image in the tops row shows double positive MuSC, the bottom row shows a MuSC that is positive for Pax7-ZsGreen but negative for Td-Tomato. Scale bar is 100 μm. (**b**) The number of MuSC were counted. More than 92% were positive for Pax7-ZsGreen and Td-Tomato, indicating a successful recombination event.

**
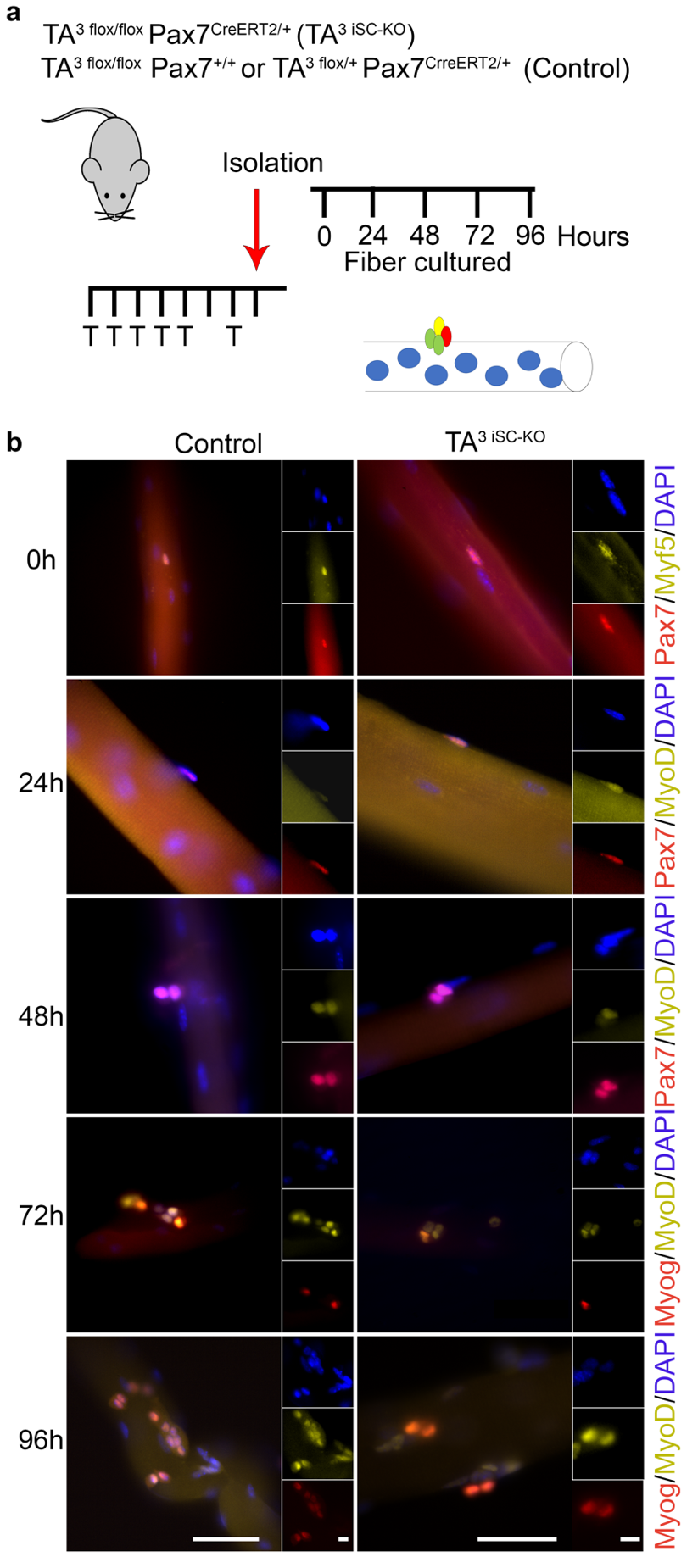
Supplementary Figure 2:** **Immunostaining of MuSC on *ex vivo* cultured myofibers with different marker genes.** (**a**) Schematic representation of the experimental design. (**b**) Representative images of EDL muscle fibers from control and TA^3iSC-KO^ mice expressing markers characteristic for each stage of the regenerative cycle (scale bars = 50 µm or 10 μm in the higher magnification images).

**
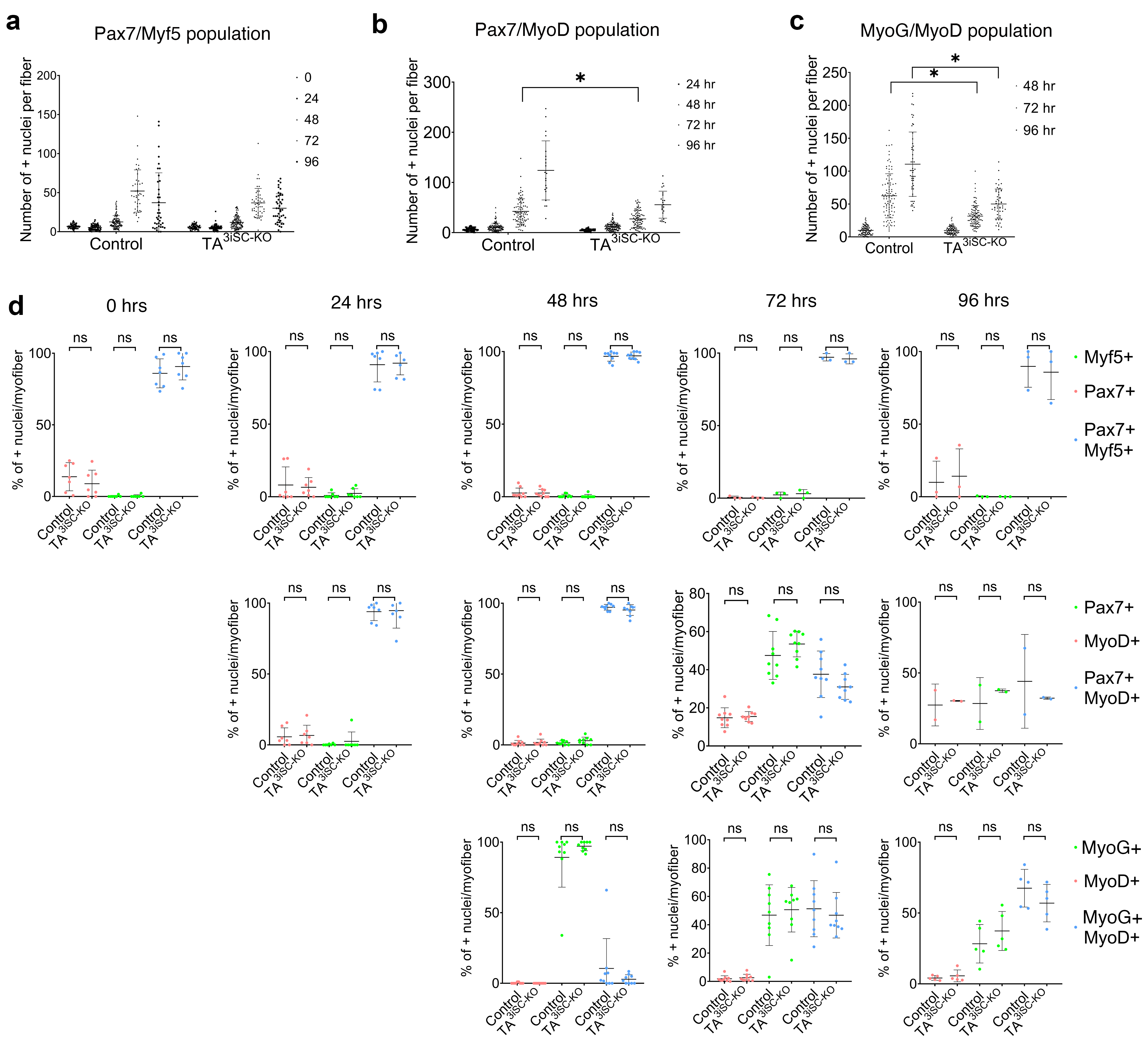
**

**Supplementary Figure 3: Quantification of MuSC immunostaining on *ex vivo* cultured myofibers.** (**a**) Total number of cells that express Pax7 and/or Myf5, (**b**) total number of cells that express Pax7 and/or MyoD, (**c**) total number of cells that express MyoG and/or MyoD at the time points indicated, in controls and TA^3iSC-KO^, n=9. *p<0.05 (**d**) Percentage of cells that are positive for one or both of the markers indicated, at the different time points indicated, in myofibers from controls and TA^3iSC-KO^ mice, n≥3 mice, unpaired, two-tailed t-test, ns, not significant, mean and SD are shown.

**
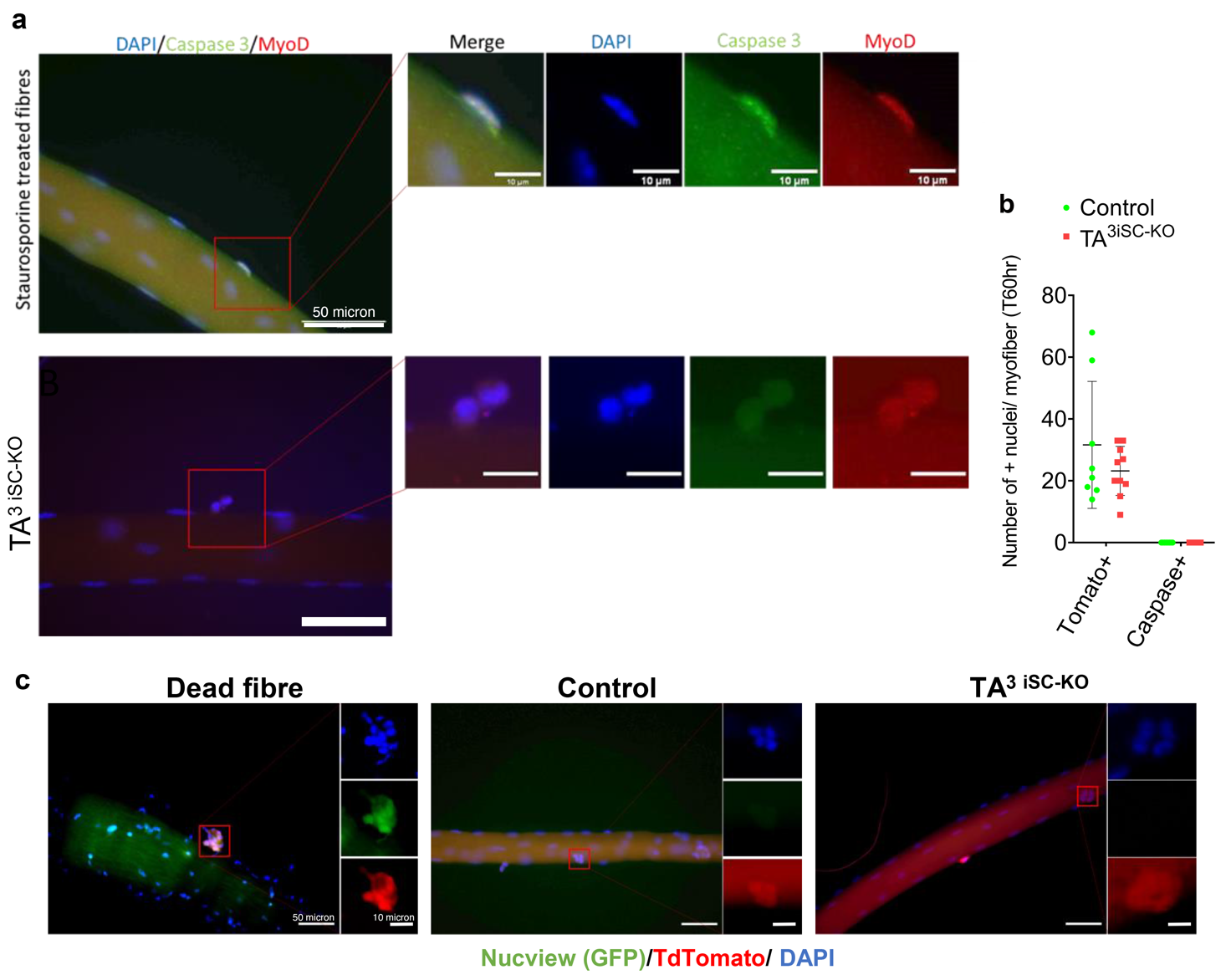
**

**Supplementary Figure 4: Caspase 3 staining shows there is no increase in apoptosis in mutant MuSC.** (**a**) Merged and single channel images are shown for DAPI stained nuclei (blue), Caspase3 (green) and MyoD (red) positive cells. Top row: as a positive control myofibers were treated with Staurosporine to induce cell death in MuSC. Bottom row shows that there were no Caspase3 positive cells; fibers from TA^3iSC-KO^ mice are shown as an example. (**b**) Number of Td-Tomato positive MuSC per myofiber, a reduced number of MuSC was detected in TA^3iSC-KO^ fibers after 60 hours (T60). No Caspase positive nuclei were observed. n = 1 mouse per genotype, 10 fibers each (**c**) Nucview was used to stain a cluster of dying cells on a necrotic fiber, as a positive control. Nucview did not detect any positive cells on control or TA^3iSC-KO^ myofibers. Scale bars are 5­­­0 μm, or 10 μm in the higher magnification images.

**
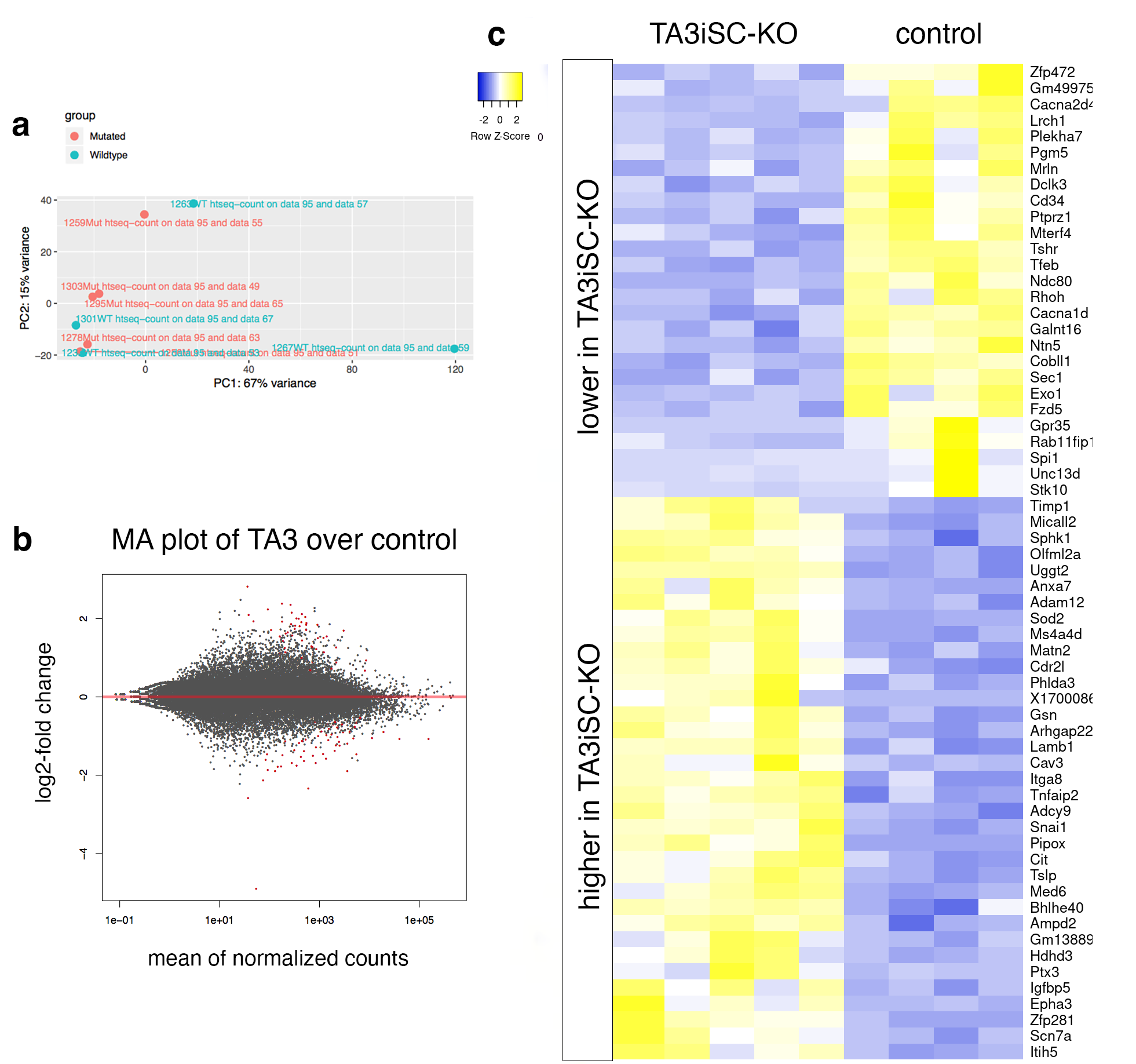
**

**Supplementary Figure 5: Pax7-ZsGreen positive MuSCs isolated by FACS from control and TA^3iSC-KO^ mice have similar molecular profiles immediately following deletion.** (**a**) PCA plots of RNAseq samples from control (blue) and TA^3iSC-KO^ MuSCs (red). (**b**) MA plots showing log2-fold change of differentially expressed (DE) genes. (**c**) Heatmap of DE genes, p-value <0.05.

**
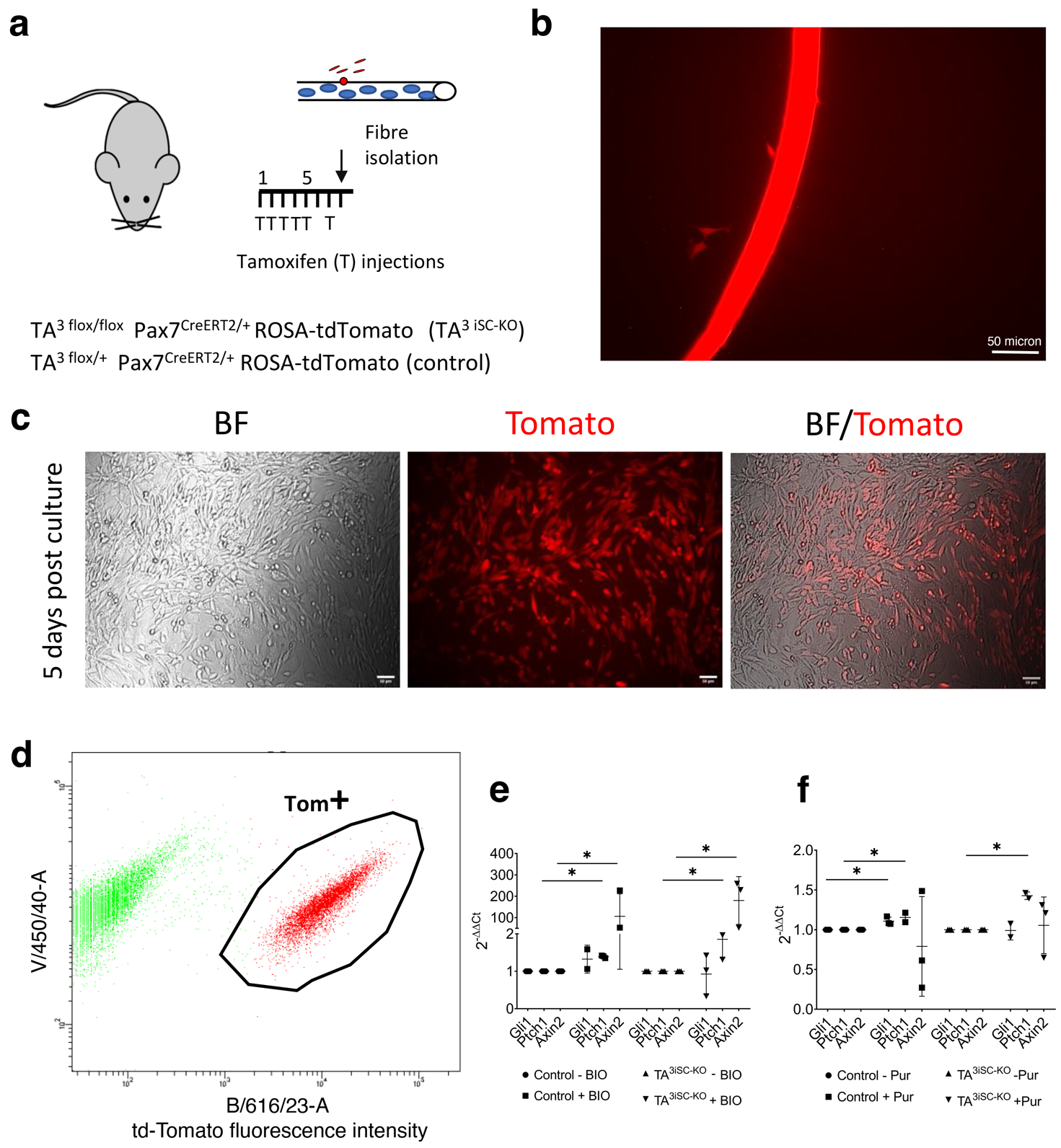
**

**Supplementary Figure 6. FACS isolation of MuSCs.** (**a**) Schematic representation of the experimental design. (**b**) Td-Tomato positive MuSC from cultured fibers grow out on matrigel coated plates. Myofibres are red due to fusion of MuSC during the regenerative cycle, scale bar 50 μm. (**c**) MuSC progeny expanding prior to drug treatments and FACS, BF, bright field; Td-Tomato fluorescence and a merged image are shown, scale bar 50 μm. (**d**) Scatter plot showing Td-Tomato positive cells (B/616/23 channel) and V450/40 fluorescence for improved separation. (**e**) qRT-PCR shows fold change of expression for Gli1, Ptch1 and Axin2, in FACS isolated MuSC progeny from control or TA^3iSC-KO^ with or without BIO, as indicated, *p ≤ 0.05 calculated by unpaired, two-tailed t-test, n=3 mice. (**f**) qRT-PCR shows fold change of expression for Gli1, Ptch1 and Axin2, in FACS isolated MuSC progeny from control or TA^3iSC-KO^ with or without Purmorphamine (Pur)­­, as indicated, *p ≤ 0.05 calculated by unpaired, two-tailed t-test, n=3 mice.

**Supplementary Table 1. Primers and protocols for mouse Genotyping.**

| **Locus** | **Primers** | **Protocol** |
| --- | --- | --- |
| Talpid3 | Forward 5’-TGCCATGCAGGGATCATAGC-3’ | 1 x 95 ⁰C 2min  10 x 95 ⁰C 30sec  68 - 58⁰C 30 sec  72 ⁰C 30sec  25 x 95 ⁰C 30sec  58 ⁰C 30sec  72 ⁰C 30sec  1 x 72 ⁰C 2min |
|  | Reverse 5’-GCTAGTACATTGCTGCAAGC-3’ |  |
|  | Null Reverse 5’-GAGCACACTGGAGGAAAGC-3’ |  |
| Pax7-cre | Forward 5’-ATCCGAAAAGAAAACGTTGA-3’ |  |
|  | Reverse 5’-AAGACCGCGAAGAGTTTGTC-3’ |  |
| Pax7-ZsGreen | Forward 5’ -CTGCATGTACCACGACTCCA- 3’ |  |
|  | Reverse 5’ -GTCAGGTGCCACTTCTGGTT- 3’ |  |
| ROSA26-TdTomato | Forward mutant 5’ -AAGGGAGCTGCAGTGGAGTA- 3’ |  |
|  | Reverse mutant 5’ -CCGAAAATCTGTGGGAAGTC- 3’ |  |
|  | Forward Wild Type 5’ -GGCATTAAAGCAGCGTATCC- 3’ |  |
|  | Reverse Wild Type 5’ -CTGTTCCTGTACGGCATGG- 3’ |  |
| Axin2_Fw | AGCAGCTCAGCAAAAAGGGA |  |
| Axin2_Rv | TTCGTACATGGGGAGCACTG |  |
| Gli1_Fw | TCTCCCTTTCTTGAGGTTGGG |  |
| Gli1_Rv | AACATGGCGTCTCAGGGAAG |  |
| Ptch1_Fw | AATTCTCGACTCACTCGTCCA |  |
| Ptch1_Rv | CTCCTCATATTTGGGGCCTT |  |
| Hprt_Fw | CATAACCTGGTTCATCATCGC |  |
| Hprt_Rv | TCCTCCTCAGACCGCTTTT |  |
