# Supplementary material for: Absence of the primary cilia formation gene Talpid3 impairs muscle stem cell function
Source: Commun Biol. 2023 Nov 4;6:1121. doi: 10.1038/s42003-023-05503-9 (PMC10625638; doi:10.1038/s42003-023-05503-9)
Supplement: Supplementary file 2 — Description of Additional Supplementary Files [file 42003_2023_5503_MOESM2_ESM.pdf]

Description of Additional Supplementary Files

File name: Supplementary Data 1
Description: All source data underlying the graphs presented in the main figures were uploaded as **Supplementary Data** (in Excel). The excel files were consolidated as separate tabs in one Excel file, identified by figure number and panel id.

File name: Supplementary Data 2
Description: All source data underlying the graphs presented in the supplementary figures were uploaded as **Supplementary Data** (in Excel). The excel files were consolidated as separate tabs in one Excel file, identified by supplementary figure number and panel id.
