# Supplementary material for: Absence of the primary cilia formation gene Talpid3 impairs muscle stem cell function
Source: Commun Biol. 2023 Nov 4;6:1121. doi: 10.1038/s42003-023-05503-9 (PMC10625638; doi:10.1038/s42003-023-05503-9)
Supplement: Supplementary file 5 — Reporting Summary [file 42003_2023_5503_MOESM5_ESM.pdf]

nature portfolio

Corresponding author(s): Andrea Munsterberg, Ulrike Mayer

Last updated by author(s): Oct 23, 2023

Reporting Summary

Nature Portfolio wishes to improve the reproducibility of the work that we publish. This form provides structure for consistency and transparency in reporting. For further information on Nature Portfolio policies, see our Editorial Policies and the Editorial Policy Checklist.

Statistics

For all statistical analyses, confirm that the following items are present in the figure legend, table legend, main text, or Methods section.

n/a	Confirmed	
☐	☒	The exact sample size (*n*) for each experimental group/condition, given as a discrete number and unit of measurement
☐	☒	A statement on whether measurements were taken from distinct samples or whether the same sample was measured repeatedly
☐	☒	The statistical test(s) used AND whether they are one- or two-sided<br>*Only common tests should be described solely by name; describe more complex techniques in the Methods section.*
☒	☐	A description of all covariates tested
☒	☐	A description of any assumptions or corrections, such as tests of normality and adjustment for multiple comparisons
☐	☒	A full description of the statistical parameters including central tendency (e.g. means) or other basic estimates (e.g. regression coefficient) AND variation (e.g. standard deviation) or associated estimates of uncertainty (e.g. confidence intervals)
☐	☒	For null hypothesis testing, the test statistic (e.g. *F*, *t*, *r*) with confidence intervals, effect sizes, degrees of freedom and *P* value noted<br>*Give P values as exact values whenever suitable.*
☒	☐	For Bayesian analysis, information on the choice of priors and Markov chain Monte Carlo settings
☒	☐	For hierarchical and complex designs, identification of the appropriate level for tests and full reporting of outcomes
☒	☐	Estimates of effect sizes (e.g. Cohen's *d*, Pearson's *r*), indicating how they were calculated

Our web collection on statistics for biologists contains articles on many of the points above.

Software and code

Policy information about availability of computer code

Data collection	no software used
Data analysis	Graphpad Prism (9.4), ggplot2, R-Studio (4.1.2), Seurat (version 3), Fiji/ImageJ for fiber diameters

For manuscripts utilizing custom algorithms or software that are central to the research but not yet described in published literature, software must be made available to editors and reviewers. We strongly encourage code deposition in a community repository (e.g. GitHub). See the Nature Portfolio guidelines for submitting code & software for further information.

Data

Policy information about availability of data

All manuscripts must include a data availability statement. This statement should provide the following information, where applicable:

- Accession codes, unique identifiers, or web links for publicly available datasets
- A description of any restrictions on data availability
- For clinical datasets or third party data, please ensure that the statement adheres to our policy

For bulk and single cell transcriptomics, the raw sequencing data can be accessed on the NCBI-SRA archive under accession number BioProject PRJNA981098. Other raw data, cell counts and myofiber diameters, are available as supplementary data files for the main (Supplementary Data 1) and supplementary figures (Supplementary Data 2).

Research involving human participants, their data, or biological material

Policy information about studies with human participants or human data. See also policy information about sex, gender (identity/presentation), and sexual orientation and race, ethnicity and racism.

Reporting on sex and gender	*Use the terms sex (biological attribute) and gender (shaped by social and cultural circumstances) carefully in order to avoid confusing both terms. Indicate if findings apply to only one sex or gender; describe whether sex and gender were considered in study design; whether sex and/or gender was determined based on self-reporting or assigned and methods used.*<br>*Provide in the source data disaggregated sex and gender data, where this information has been collected, and if consent has been obtained for sharing of individual-level data; provide overall numbers in this Reporting Summary. Please state if this information has not been collected.*<br>*Report sex- and gender-based analyses where performed, justify reasons for lack of sex- and gender-based analysis.*
Reporting on race, ethnicity, or other socially relevant groupings	*Please specify the socially constructed or socially relevant categorization variable(s) used in your manuscript and explain why they were used. Please note that such variables should not be used as proxies for other socially constructed/relevant variables (for example, race or ethnicity should not be used as a proxy for socioeconomic status).*<br>*Provide clear definitions of the relevant terms used, how they were provided (by the participants/respondents, the researchers, or third parties), and the method(s) used to classify people into the different categories (e.g. self-report, census or administrative data, social media data, etc.)*<br>*Please provide details about how you controlled for confounding variables in your analyses.*
Population characteristics	*Describe the covariate-relevant population characteristics of the human research participants (e.g. age, genotypic information, past and current diagnosis and treatment categories). If you filled out the behavioural & social sciences study design questions and have nothing to add here, write "See above."*
Recruitment	*Describe how participants were recruited. Outline any potential self-selection bias or other biases that may be present and how these are likely to impact results.*
Ethics oversight	*Identify the organization(s) that approved the study protocol.*

Note that full information on the approval of the study protocol must also be provided in the manuscript.

Field-specific reporting

Please select the one below that is the best fit for your research. If you are not sure, read the appropriate sections before making your selection.

☒ Life sciences ☐ Behavioural & social sciences ☐ Ecological, evolutionary & environmental sciences

For a reference copy of the document with all sections, see nature.com/documents/nr-reporting-summary-flat.pdf

Life sciences study design

All studies must disclose on these points even when the disclosure is negative.

Sample size	No tests were used to predetermine sample size. Sample size was based on previous experience using mouse muscle stem cells, for example Palla, A.R. et al. Nat Commun 13, 1439 (2022).
Data exclusions	For histological analysis of Feret (myofiber) diameters, we excluded fibers that did not have centrally located nuclei.<br>For single cell sequencing, Myofibers were cultured ex vivo. After 72 hours Td-Tomato fluorescent MuSCs were isolated by FACS, sorted into plates (2x3=6 96-well plates per genotype, 576 wells per genotype) and sequenced using Smart-Seq2. From three independent biological replicate samples each, for control and TA3iSC-KO MuSC, we retained 832 MuSC after quality control. The cell numbers per cluster are detailed in the results.
Replication	A minimum of three independent experiments (or animals) were used for all assays. For each experimental approach, control and mutant animals were treated on the same day. The exact number of replicates in independent experiments is mentioned in the figure legends. For the scRNAseq we collected by FACS the MuSCs from one control and one mutant animal per day and sorted into plates. This was done 3 times independently and all data from the 3 biological replicates was pooled.
Randomization	After genotyping mice were given a numerical ID and allocated to their experimental groups. There was no randomization.
Blinding	Investigators were using the numerical ID allocated during the subsequent analysis, e.g. cryosections for histology, myofiber isolation for immunostaining and cell counts. The genotype or treatment was decoded after image acquisition and analysis.

Reporting for specific materials, systems and methods

We require information from authors about some types of materials, experimental systems and methods used in many studies. Here, indicate whether each material, system or method listed is relevant to your study. If you are not sure if a list item applies to your research, read the appropriate section before selecting a response.

Materials & experimental systems

n/a	Involved in the study	
☐	☒	Antibodies
☒	☐	Eukaryotic cell lines
☒	☐	Palaeontology and archaeology
☐	☒	Animals and other organisms
☒	☐	Clinical data
☒	☐	Dual use research of concern
☒	☐	Plants

Methods

n/a	Involved in the study	
☒	☐	ChIP-seq
☐	☒	Flow cytometry
☒	☐	MRI-based neuroimaging

Antibodies

Antibodies used	Primary antibodies against Pax7 and Myogenin are purified from hybridoma supernatants (DSHB), Myf5 (SC-302), MyoD (SC-304) (Santa Cruz), Arl13B (Proteintech, 1:300). Nidogen1 (Willem et al., 2002, ref. 71), Caspase-3 (Asp175) (Cell Signalling 9661). Secondary antibodies used were goat anti-mouse IgG-Fab2 488 and goat anti-rabbit Alexa fluor 546 (1:375) (Jacksonimmuno). Anti-wheat germ agglutinin (WGA) conjugated with Alexa Fluor 488 (Invitrogen W11261) (1:200).
Validation	All primary antibodies are validated for detection of the protein of interest according to information on the manufacturers website. Details of the validation statement, antibody profiles and relevant citations can be found on the manufacturer website or in the cited publications.

Animals and other research organisms

Policy information about studies involving animals; ARRIVE guidelines recommended for reporting animal research, and Sex and Gender in Research

Laboratory animals	C57Bl6J mice (Jackson laboratory) were use between 8-12 weeks old.
Wild animals	No wild animals were used.
Reporting on sex	Sex was not considered in this study and both male and female animals were included.
Field-collected samples	No field collected samples were included.
Ethics oversight	Mice were kept in controlled conditions on 12:12 light:dark cycle, water and food ad libitum. Experiments are regulated by the Animals (Scientific Procedures) act 1996 and conducted following ethical review by the University of East Anglia's Animal and Ethical Review body (AWERB). Experiments were approved by the Home Office Project Licenses 70/8824 and PP3253888. This information is provided in the Methods section of the manuscript.

Note that full information on the approval of the study protocol must also be provided in the manuscript.

Plants

Seed stocks	*Report on the source of all seed stocks or other plant material used. If applicable, state the seed stock centre and catalogue number. If plant specimens were collected from the field, describe the collection location, date and sampling procedures.*
Novel plant genotypes	*Describe the methods by which all novel plant genotypes were produced. This includes those generated by transgenic approaches, gene editing, chemical/radiation-based mutagenesis and hybridization. For transgenic lines, describe the transformation method, the number of independent lines analyzed and the generation upon which experiments were performed. For gene-edited lines, describe the editor used, the endogenous sequence targeted for editing, the targeting guide RNA sequence (if applicable) and how the editor was applied.*
Authentication	*Describe any authentication procedures for each seed stock used or novel genotype generated. Describe any experiments used to assess the effect of a mutation and, where applicable, how potential secondary effects (e.g. second site T-DNA insertions, mosiacism, off-target gene editing) were examined.*

Flow Cytometry

Plots

Confirm that:

☒ The axis labels state the marker and fluorochrome used (e.g. CD4-FITC).

☒ The axis scales are clearly visible. Include numbers along axes only for bottom left plot of group (a 'group' is an analysis of identical markers).

☒ All plots are contour plots with outliers or pseudocolor plots.

☐ A numerical value for number of cells or percentage (with statistics) is provided.

Methodology

Sample preparation	For RNAseq, hindlimb muscles were minced and digested using a solution of collagenase type 2 (Worthington Biochemicalm cat No. LS004177) and dispase II (Roche). Homogenized muscles were passed through 100, 40 and 20 micron nylon strainers to obtain a single cell suspension. The cells were subjected to FAC sorting and pooled for bulk RNA sequencing.<br>For scRNAseq cells were directly sorted into 96-well dishes. For RT-PCR, MuSCs were grown out from isolate myofibers and FAC sorted based on Td-Tomato fluorescence.
Instrument	BD FACS ARIA II
Software	FACSDIVA Software (BD Bioscience)
Cell population abundance	Cells were not post-sorted after initial purity sorting.
Gating strategy	Fluorescent muscle stem cells were sorted from muscle tissue, or outgrown MuSCs from fibers:<br>FSC and SSC were used to identify cell and to exclude debris. After gating for live cells using Zs-Green (RNA transcriptomics) or Td-Tomato (RT-PCR and scRNAseq), cells negative for these markers were excluded. Td-Tomato positive cells (B/616/23 channel) and V450/40 fluorescence for improved separation

☒ Tick this box to confirm that a figure exemplifying the gating strategy is provided in the Supplementary Information.
